# Fitness Effects of Mutations: An Assessment of PROVEAN Predictions Using Mutation Accumulation Data

**DOI:** 10.1093/gbe/evac004

**Published:** 2022-01-17

**Authors:** Linnea Sandell, Nathaniel P Sharp

**Affiliations:** Department of Zoology, University of British Columbia, Vancouver, British Columbia, Canada; Systematic Biology, Department of Organismal Biology, Uppsala University, Sweden; Department of Genetics, University of Wisconsin—Madison, USA

**Keywords:** distribution of fitness effects, ecological genetics, essential genes, *Saccharomyces cerevisiae*, *Chlamydomonas reinhardtii*

## Abstract

Predicting fitness in natural populations is a major challenge in biology. It may be possible to leverage fast-accumulating genomic data sets to infer the fitness effects of mutant alleles, allowing evolutionary questions to be addressed in any organism. In this paper, we investigate the utility of one such tool, called PROVEAN. This program compares a query sequence with existing data to provide an alignment-based score for any protein variant, with scores categorized as neutral or deleterious based on a pre-set threshold. PROVEAN has been used widely in evolutionary studies, for example, to estimate mutation load in natural populations, but has not been formally tested as a predictor of aggregate mutational effects on fitness. Using three large published data sets on the genome sequences of laboratory mutation accumulation lines, we assessed how well PROVEAN predicted the actual fitness patterns observed, relative to other metrics. In most cases, we find that a simple count of the total number of mutant proteins is a better predictor of fitness than the number of proteins with variants scored as deleterious by PROVEAN. We also find that the sum of all mutant protein scores explains variation in fitness better than the number of mutant proteins in one of the data sets. We discuss the implications of these results for studies of populations in the wild.

Significance

PROVEAN is a widely used bioinformatic tool to summarize the health of different populations based on their mutations, but there have been no attempts at verifying its predictions on a genome level. We test how well this program can explain differences in growth rate among populations of yeast and green algae. We find that counting the total number of mutant proteins, without considering the program’s predictions, does better at explaining differences in growth rate in two out of three data sets. This means researchers might lose information of population differences when using this tool. There need to be more tests of the usefulness of these scores in an eco-evolutionary setting.

## Introduction

In a well-adapted population, mutations that are deleterious will tend to be eliminated by selection, whereas neutral variants may persist. When we compare similar proteins from different species, we find that variation is unequally distributed across the amino acid sequence: some sites are highly variable whereas others are constrained. A lack of variation at some sites among homologous protein sequences is believed to reflect a history of purifying selection against deleterious mutations. A history of selection also implies that most new mutations will be neutral or deleterious, with rare exceptions, because available beneficial changes have already appeared and become fixed. This framework leads to the prediction that the frequency of an allele among homologous sequences should be inversely proportional to its deleterious effect on fitness (Ohta 1992). Several programs have been developed to identify deleterious alleles based on this general framework, and their utility has been validated based on known disease-causing alleles in humans (Ng and Henikoff 2001, 2006).

Predicting the impact of amino acid substitutions on protein function and fitness has always been a key goal of molecular evolution research. An early approach compared proteins among related species to determine which amino acids were most substitutable over evolutionary time. Highly similar proteins were gathered, and the frequency of different amino acid substitutions in these sequences were used to determine a substitution matrix. Henikoff and Henikoff (1992) gathered conserved regions of proteins (blocks) from the PROSITE database for over 500 protein families to generate BLOSUM—the Blocks Substitution Matrix. The score for a certain amino acid substitution in the BLOSUM is a log-odds ratio, log(*H*_1_/*H*_0_), where *H*_0_ is the probability of seeing the two amino acids align by chance (frequency of amino acid one times the frequency of amino acid two) and *H*_1_ is the frequency of this amino acid pair in the conserved blocks (for a primer on BLOSUM, see Eddy [2004]).

Cargill et al. (1999) used the BLOSUM62 matrix to rank single-nucleotide polymorphisms in human proteins as conserved or not. This spurred Ng and Henikoff (2001) to develop SIFT (Separate Intolerant From Tolerant); they argued that the functional impact of an amino acid substitution cannot be solely predicted by its score in the BLOSUM matrix. Rather, one needs to consider the position in the sequence and existing polymorphisms in highly homologous sequences. Although BLOSUM62 is based on conserved regions of highly diverged proteins, SIFT relies on closely related proteins to predict whether a mutation exists in a functionally important region, and calculates the probability of substitution for each position. For any given mutant protein, SIFT can predict whether it will be deleterious given its probability in the site-specific matrix.

Concurrently with the development of SIFT, Adzhubei et al. (2010) presented PolyPhen-2 which, in contrast to their predecessor PolyPhen (Ramensky et al. 2002) and SIFT, combines multiple predictive features, including structure-based predictors such as the accessible surface area of the protein, as well as multiple sequence alignment. Other tools that can be used on nonhuman variants (but in contrast to PolyPhen-2 and SIFT are not relying on predefined alignments) include MAPP (Stone and Sidow 2005), GERP++ (Davydov et al. 2010), and likelihood ratio tests (Chun and Fay 2011). A lot of focus has been put on evaluating and comparing the different tools on testing data sets (Dong et al. 2015; Grimm et al. 2015; Kono et al. 2018). Those studies have evaluated the tools’ predictive power on individual protein changes, not on the aggregate effect of multiple proteins with variants. We are interested in what benefit these kinds of tools can offer to the eco-evolutionary researcher for quantifying the genetic health of their (nonhuman) population of study, similar to the studies done on the cost of domestication (Renaut and Rieseberg 2015; Kono et al. 2016; Moyers et al. 2018).

This paper focuses solely on the Protein Variant Effect Analyzer (PROVEAN), introduced by Choi et al. (2012) to predict the effects of not only amino acid substitutions but also in-frame insertions and deletions. The field of computational variant effect prediction is under continual development, and PROVEAN is not necessarily the most widely used tool in all disciplines. However, PROVEAN has come to be a very popular tool in genomic inference in ecology and evolution and has been cited in almost 2,000 research articles as of November 2021 (Web of Science citation report). Though PROVEAN was developed with and evaluated on human medical data it has increasingly been used to categorize polymorphisms in a variety of nonhuman species (supplementary table S1, Supplementary Material online). Notably, eco-evolutionary studies have applied PROVEAN in comparisons of populations that differ not in one, but many, proteins. Many studies use PROVEAN to draw conclusions about relative mutation load, for example, in different habitats of nocturnal lemurs (Veilleux et al. 2013), between native and invasive species of Asteraceae (Hodgins et al. 2015), between domesticated and wild Compositae crops (Renaut and Rieseberg 2015), populations of Lake Trout (Perrier et al. 2017), and between new sex chromosomes in cichlids (Gammerdinger et al. 2019).

Although our focus on PROVEAN specifically was based on the considerations above, there is overlap between this method and other programs, most particularly SIFT and PolyPhen-2, which use sequence comparisons from BLAST searches and therefore are sensitive to the choice of database (Choi et al. 2012; Kono et al. 2018). Similar to SIFT, PROVEAN gathers clusters of highly homologous sequences in the NCBI nonredundant protein sequences (nr) database. Rather than producing probabilities of substitution across the protein of interest, PROVEAN computes an alignment score for both the query sequence (i.e., wild type) and the mutant to these sequence clusters. The difference in the mean alignment score for the query and mutant protein is called the PROVEAN score. The BLOSUM62 matrix that is used for protein alignment in PROVEAN has blocks aligned from proteins that are less than 62% identical. A 62% cutoff ensures that the proteins that are being compared are divergent; within these proteins, only the conserved regions are used in the BLOSUM matrix, ensuring that their similarities and differences reflect selection, or a lack thereof.

**Fig. 1.**
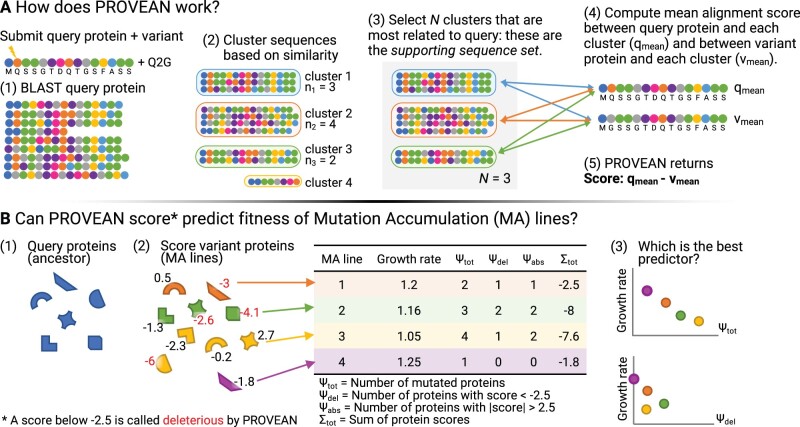
Conceptual figure of project. (*A*) Outline of PROVEAN process, inspired by figure 10 in Choi et al. (2012). (*B*) The shapes in the bottom left represent different proteins within the ancestor. Each MA line (represented by color) has some number of protein(s) mutated compared with the ancestor. The same protein can have different mutations in different MA lines, causing the score given by PROVEAN to differ (the variant of the rainbow protein carried by the orange MA line is given a score 0.5, whereas the variant of the rainbow protein carried by the yellow line receives a score of −0.2). All proteins with variants between the MA ancestor and MA lines were submitted to PROVEAN. Each MA line had the number and PROVEAN scores of their mutant proteins summarized (table). Finally, we looked for correlations between the fitness of MA lines and their number of mutant proteins (top graph in 3) or the number of mutant proteins scored as deleterious by PROVEAN (bottom graph in 3).

In the first step of PROVEAN, a BLAST (Altschul et al. 1990; Camacho et al. 2009) search is done using the submitted query sequence. An Expect (E) value cutoff of 0.1 is used to find homologous, while still distantly related, sequences. This typically results in thousands of matches across a wide range of taxa. To avoid redundancy these sequences are clustered based on a cutoff of 75% sequence similarity within a cluster. Then, the top 30 clusters most similar to the query sequence are used to calculate alignment scores to the query and mutant sequence and finally the PROVEAN score. The supporting sequence set can be saved and analyzed independently. The PROVEAN process is summarized in figure 1*A* (inspired from figure 10 in Choi et al. [2012]).

Based on the PROVEAN score, the program reports a predicted functional category, either deleterious or neutral, based on a pre-set threshold. Though it is possible for a mutant protein to receive a higher mean alignment score than the wild type, there is no category for beneficial effects. The default threshold value is −2.5, and variants with scores below this threshold are classified as deleterious. This threshold was chosen to maximize sensitivity (detection) and specificity (accuracy) when assigning functional effects to common versus disease-causing human protein variants (Choi et al. 2012; Choi and Chan 2015).

The creators of PROVEAN suggest adjusting the threshold score for defining deleterious alleles depending on the user’s needs. However, in the studies summarized in supplementary table S1, Supplementary Material online, that applied PROVEAN to nonhuman organisms, nearly all used the default cutoff value. Although there have been studies evaluating different methods of functional annotation of mutations, they do not consider the possibility of species-specific thresholds (Kono et al. 2018). How well does this tool, which was developed with data from humans, work in predicting fitness effects in other organisms, when many variants are present?

Experimental systems offer an opportunity to evaluate the explanatory power of PROVEAN predictions in nonhuman systems, but such research has been limited. Lind et al. (2010, 2016) measured the individual fitness effects of 156 mutagenesis-induced mutations in specific genes in *Salmonella*. They found that PROVEAN scores correlated well with the fitness effects of mutations in three genes required for growth on arabinose. In contrast, PROVEAN scores for mutations in two ribosomal proteins did not significantly correlate with fitness effects. Our aim was to take a similar approach using larger samples of random mutations, with multiple mutations found on each genetic background, similar to many instances where PROVEAN is applied.

Mutation accumulation (MA) experiments attempt to separate mutation from selection by repeatedly bottlenecking replicate populations so that drift rather than selection dominates the probability that a mutation establishes within each lineage (Halligan and Keightley 2009). With a nearly random sample of mutations in many MA lines, we can use PROVEAN to predict the impact of the mutations and compare these predictions to direct estimates of relative fitness based on laboratory assays. We can also compare how well the default parameters work to find homologous sequences in nonhuman species.

We analyzed three MA data sets that combine genomic information with growth rate data, allowing us to evaluate whether mutations scored as deleterious by PROVEAN correlate with measured fitness (fig. 1*B*): *Saccharomyces cerevisiae* data set 1 (Sc1) (Sharp et al. 2018), *S. cerevisiae* data set 2 (Sc2) (Liu and Zhang 2019), and *Chlamydomonas reinhardtii* (Cr) (Ness et al. 2015; Kraemer et al. 2017). These data sets span different kingdoms, ploidy levels (Sc1), environments (Sc2), and genetic backgrounds (Cr). We assessed the predictive value of this tool by asking whether incorporating PROVEAN scores in a regression model provides a better fit to MA line fitness data than a model that simply predicts fitness based on the number of mutant proteins.

The PROVEAN method implicitly assumes that large changes to proteins are deleterious (the more dissimilar to the query sequence the worse the score). Adaptation to a new environment requiring significant and novel changes to a protein could thus be misclassified as deleterious. Some studies, for example, Yoshida et al. (2016) and Kusakabe et al. (2017) have used the absolute PROVEAN score to find function-altering alleles, thereby including variants with scores substantially above or below zero, and so we also considered this approach in some of our tests.

## Results

In each MA data set, we examined the relationship between relative fitness and the number of mutant proteins, Ψ. Our key question is whether the number of variant proteins scored as “deleterious” by PROVEAN, Ψ_del_, is a better predictor of fitness than the total number of variant proteins, Ψ_tot_, which does not incorporate information from PROVEAN. We assume that the ancestor of a given set of MA lines has Ψ_tot_=Ψ_del_=0 by definition, and a relative fitness value of 0, such that the model intercepts can be fixed at (0, 0). By incorporating the MA controls in this way, we increase power to detect effects of mutant proteins on fitness generally, but we later explore whether this approach affects our key comparisons.

In addition to these key models, we asked whether prediction can be improved by incorporating additional information from the PROVEAN scores and model systems. For data sets using *S. cerevisiae*, where the “essentialness” of each gene has been determined (Winzeler et al. 1999), we assessed the predictive value of variant proteins in essential genes only: Ψ_tot_ess_ and Ψ_del_ess_. We also considered the number of variant proteins with absolute PROVEAN scores above 2.5 as a possible predictor (Ψ_abs_, Ψ_abs_ess_). The distinction between the Ψ_del_ and the Ψ_abs_ metrics is that the former counts mutant proteins with scores below a certain threshold, whereas the latter counts mutant proteins with an absolute value of their score bigger than the absolute value of the chosen threshold. If the default threshold of −2.5 was used, a mutant protein that received a score of +3 would be included in Ψ_abs_ but not in Ψ_del_. There was no score above +2.5 in the haploid lines of Sc1, which means Ψ_del_=Ψ_abs_ and Ψ_del_ess_=Ψ_abs_ess_. In Sc2 there was no score above +2.5 in the essential genes such that Ψ_del_ess_=Ψ_abs_ess_. Finally, we examined whether the log-transformed sum of all PROVEAN scores was a predictor of fitness (∑_tot_ and ∑_tot_ess_).

**Fig. 2.**
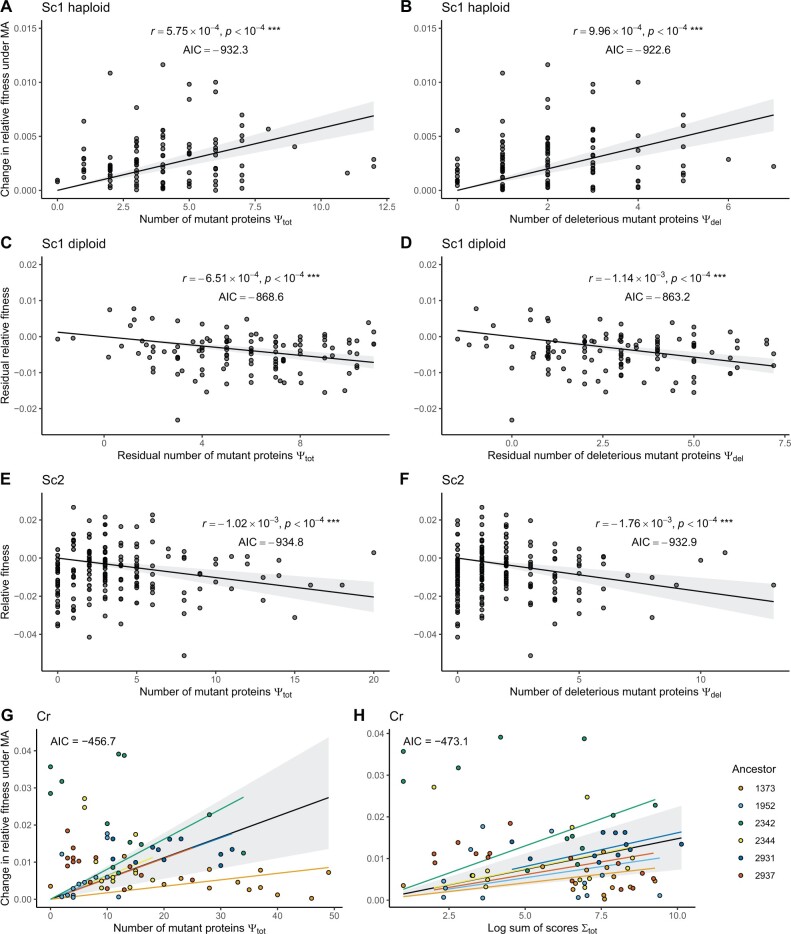
Partitioning mutations based on their PROVEAN score does not improve our models in yeast (compare *A* with *B*, *C* with *D*, and *E* with *F*). In the *Chlamydomonas reinhardtii* data set, the best model uses the sum of PROVEAN scores (compare *G* with *H*). Each dot represents one mutation accumulation line. Ancestral controls are not shown. Gray bands are 95% confidence intervals. In *G* and *H*, the black line and gray band show the overall effect of the explanatory variable, and the colored lines show the random slope for each ancestor.

In all three data sets, we find that both Ψ_tot_ and Ψ_del_ are significant predictors of fitness (fig. 2). But our main interest is in whether PROVEAN adds explanatory power, above and beyond a simpler alternative. For the most part, we find that models with Ψ_tot_ as a predictor are preferred over models incorporating PROVEAN scores, based on Akaike’s information criterion (AIC) scores (fig. 2). However, in the Cr data set, the sum of PROVEAN scores is the preferred predictor of fitness differences. The formula for AIC is 2*K*−ln(*L*), where *K* is the number of model parameters and *L* is the log-likelihood of the model. The reason why the AIC scores of our models are negative is because we have few (or only one) predictors but the log-likelihood scores of our models are large. We provide details of our model results below.

### *Saccharomyces cerevisiae* Data Set 1 (Sc1)

This data set consists of both haploid and diploid MA lines. In the haploid MA lines, no significant decline in mean fitness was detected, though genetic variation was present (Sharp et al. 2018). We therefore used the absolute change in growth rate relative to the ancestor as the response variable in linear models for the haploid lines. We found that all our explanatory variables individually are significant predictors of fitness (see Supplementary Material online), but that the model using the total number of mutant proteins Ψ_tot_ was preferred. All other models evaluated had a ΔAIC score higher than 9 compared with this model.

For the diploid MA lines, we included relative genome size as a covariate to account for the effects of aneuploidy, which we previously found shows a strong relationship with fitness in these lines (Sharp et al. 2018) (aneuploidy was not observed in haploids). These models showed the same qualitative results as in the haploids. The model using the total number of mutated proteins was preferred, and all other models had a ΔAIC of 5 or higher compared with this.

### *Saccharomyces cerevisiae* Data Set 2 (Sc2)

This data set involves strains growing in multiple environments, but we did not find an effect of environment on the fitness of MA lines relative to controls growing in the same environment. We also did not detect an effect of aneuploidy in this data set. We found that all our other explanatory variables individually were significant predictors of fitness (see Supplementary Material online), but that the model using the total number of mutant proteins Ψ_tot_ was preferred. The model using Ψ_del_ was the next best, and in contrast to the result in Sc1, there was no significant difference in AIC score between the two top models (the model using Ψ_del_ had a ΔAIC score of <2).

### *Chlamydomonas reinhardtii* Data Set (Cr)

This data set involves multiple genetic backgrounds, and so we applied linear mixed-effect models with the ancestral strain as a random effect on the slope. We found that all our explanatory variables individually were significant predictors of fitness (see Supplementary Material online), but that the model using Σ_tot_ was preferred (all other models had a ΔAIC score larger than 10), in contrast to the yeast data sets. Some genetic backgrounds show potential increases and decreases in fitness, but we obtained the same qualitative results when considering the absolute change in relative fitness as the response variable.

### Varying the PROVEAN Cutoff Value

**Fig. 3.**
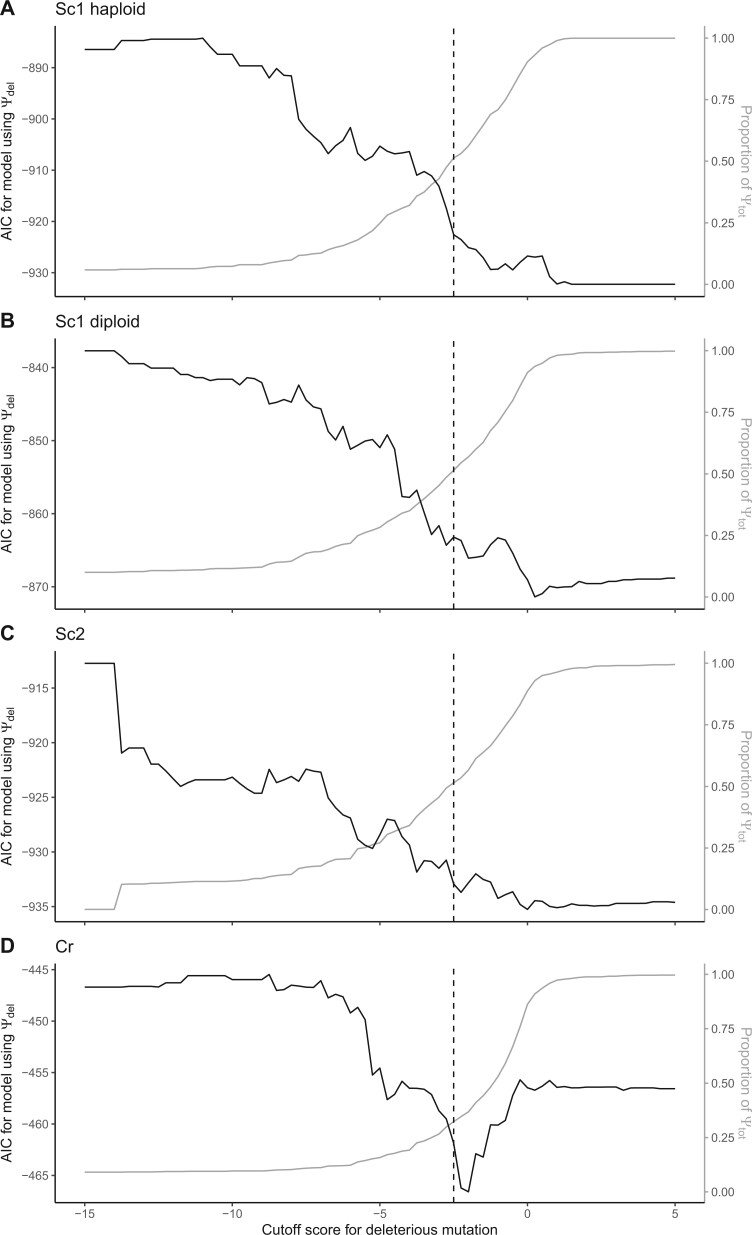
Varying the threshold for what to call deleterious mutations. The black line shows AIC score (on the left axis), and the gray line shows the proportion of mutant proteins that is considered deleterious (the right axis). In yeast (*A*, *B*, *C*), the best model uses all mutations, that is, the AIC score of the model using Ψ_del_ as the explanatory variable decreases the closer Ψ_del_ gets to Ψ_tot_. In Cr, a cutoff of approximately −2 gives Ψ_del_ the biggest ΔAIC compared with the model using all protein mutants.

As the PROVEAN creators suggest changing the threshold or cutoff value depending on the circumstance, we investigated how the AIC of our model using Ψ_del_ changes based on the cutoff value (fig. 3). The AIC score of the model declines as Ψ_del_ approaches Ψ_tot_ in both yeast data sets, that is, the more mutant proteins that are included into the model, the better the variance in fitness is explained. In contrast, the AIC score of the model in the Cr data set has a nonmonotonic relationship with the cutoff value; the lowest AIC score is achieved with a threshold of −2 (fig. 3). These results again imply that PROVEAN score has some explanatory potential above that of the total number of mutant proteins in the Cr data set but not in the yeast data sets.

### Differences in the Number of Clusters and Supporting Sequences between Species

Although the accuracy of PROVEAN is reported to be robust to the number of clusters used for scoring, there is a significant drop in accuracy for very small cluster numbers in the original study of human variants (figure S4 in Choi et al. [2012]). In addition, accuracy decreases if the number of supporting sequences chosen to make the clusters falls below 50 (figure 9 in Choi et al. [2012]); 122 of the 1,534 yeast proteins that we analyzed involved fewer than 30 clusters (8%), and 79 proteins had fewer than 50 supporting sequences (5%). Even more (13%) of the *C. reinhardtii* proteins received fewer than 30 clusters (187 out of 1,444), and three-quarters of the proteins (1,082 out of 1,444, 75%) had fewer than 50 supporting sequences. This lack of supporting sequences may reflect an underrepresentation of algal proteins in the NCBI protein database, which leads to few homologous sequences passing the E-value threshold of 0.1. This is also evident from the average E-value score of proteins, which is an order of magnitude larger in Cr than in Sc1 and Sc2 (19.9×10^−4^ compared with 1.6×10^−4^ for Sc1 and 2.9×10^−4^ for Sc2). This means that the scores for variant proteins in Cr were based on alignments to fewer sequences with lower similarity, which is expected to reduce accuracy.

### Reference Bias

For all proteins that differed between the laboratory ancestor and any MA line, we looked for differences between the laboratory ancestor and the reference genome. Although we found few mutated proteins with preexisting differences between the laboratory strain and reference genome in the two yeast data sets (142 out of 1,880 proteins), most mutated proteins in Cr already differed between the laboratory strains and the reference genome (1,136 out of 1,369, table 1). These protein differences between the MA ancestor and the reference genome most often were complex, involving more than one kind of mutation (substitution, insertion, and/or deletion). We explored how this preexisting natural variation would be scored by PROVEAN. That is, we coded the proteins with variants differentiating each ancestral laboratory strain and the reference genome and submitted these variants with the protein of the laboratory strain as the query sequence.

**Table 1:** Number of Protein-Coding Mutations for MA Lines of a Particular Ancestral *Chlamydomonas reinhardtii* Line and the Ancestral Line’s Protein-Coding Mutations

Ancestor Strain ID	Number of Mutated Proteins in MA Lines^a^	Number of which Had Prior Mutation(s) in Ancestor	Number of MA Lines	Number of Protein Variants^b^
1373	381	234	12	390
1952	80	75	14	82
2342	188	171	12	192
2344	239	225	15	244
2931	351	322	14	358
2937	130	109	15	131
Total	1,369	1,136	82	1,397

a Each protein is counted only once, even if several MA lines have mutations in the protein.

b Each change to a protein in any mutant sample is counted. This is the number of protein variants submitted to be scored by PROVEAN.

**Fig. 4.**
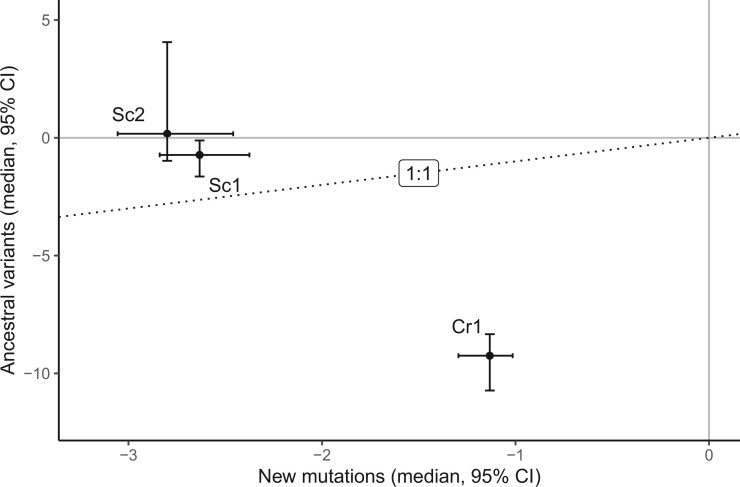
Median PROVEAN scores for new mutations and ancestral (preexisting) variants in the three data sets. Error bars are 95% confidence intervals.

Median scores for the ancestral variants and new mutations in each data set are plotted in figure 4. Unlike new mutations in MA lines, differences between the reference and ancestral genotypes have potentially been “screened” by natural selection, and we therefore expect them to be less deleterious. This prediction is supported in the two yeast data sets, where the ancestral variants were less likely than new mutations to be scored as deleterious under the default threshold (Fisher’s exact tests; Sc1: *P *=* *0.026; Sc2: *P *=* *0.026), and have less extreme (i.e., higher) PROVEAN scores overall (Wilcox tests; Sc1: *P *<* *1 × 10^−9^; Sc2: *P *<* *1 × 10^−5^). In contrast, ancestral variants in the Cr data set were more likely to be scored as deleterious than new mutations (logistic regression with random effects of ancestral strain, *P *<* *1 × 10^−15^), and had more extreme (negative) scores overall (Wilcox test for each strain background, all *P *<* *1 × 10^−8^).

To boost the predictive value of our models, we leveraged information from control lines where fitness was measured and new mutations were absent. Although such “standard” genotypes might be available in some study systems, we would ideally like to be able to detect a genotype–phenotype relationship among mutants alone, that is, by not fixing the model intercepts or incorporating information from “controls.” In the context of MA data, this approach will reduce power, particularly when the variance in the number of proteins with variants per MA line is small. We re-evaluated our linear models after removing the fixed intercept. In haploid Sc1, only the sum of scores ∑_tot_ remains a significant predictor of fitness differences among MA lines, and this performs significantly better than the corresponding model using Ψ_tot_ (ΔAIC = 2.62). In diploid Sc1 no predictor is significant when the intercept is not fixed; whereas Ψ_del_ess_ is the preferred predictor, this does not significantly outperform Ψ_tot_ (ΔAIC = 1.88) or Ψ_tot_ess_ (ΔAIC = 1.27). This pattern was also the case for the Sc2 data set (see Supplementary Material online). Similarly, in the Cr data set, predictors are not significant without a fixed intercept, and the best model uses Ψ_del_ but does not significantly outperform a model using Ψ_tot_ (ΔAIC = 0.35).

## Discussion

Predicting the impact of mutations on fitness is an important goal in molecular population genetics. Several tools implicitly assume that beneficial alleles sweep to fixation and deleterious mutations are purged, such that neither exist as common polymorphisms in standing genetic variation, whereas alleles with nearly neutral effects can be found at higher frequencies. Such differences in the site frequency spectrum are ultimately the basis for methods like PROVEAN, which is widely used in the life sciences (supplementary table S1, Supplementary Material online). Although efforts have been made to compare approaches to estimate fitness effects computationally (Huber et al. 2020; Chen et al. 2021), few studies compare the predictions with empirical fitness estimates. MA experiments offer a way to evaluate these methods, as the effect of mutations on fitness is measured in controlled environments with consistent genetic backgrounds.

We asked whether the information from PROVEAN could explain variation in growth rate in three independent sets of MA lines. In two *S. cerevisiae* data sets (Sc1 and Sc2), using PROVEAN to categorize variant proteins as deleterious or neutral did not provide improvements in model fit beyond what can be achieved by simply considering the total number of mutant proteins. In the *C. reinhardtii* (Cr) data set, incorporating PROVEAN information was useful, especially when considering the sum of the PROVEAN scores themselves.

There are several possible explanations for why PROVEAN scores do not always add useful information for predicting fitness. First, it could be that PROVEAN scores accurately delineate variants whose effects, averaged over many genetic backgrounds and many generations of evolution, are deleterious versus neutral, but that the short-term effects of protein variants are not always correlated with their long-term effects. An extreme demonstration of this was seen in the yeast knock-out project, where less than half of gene disruptions had a detectable effect on growth rate in the standard lab environment (Winzeler et al. 1999), but these genes are nevertheless maintained by selection in the long run. In the data sets we studied, about half of the proteins with variants were classified as deleterious by PROVEAN under the standard threshold; the fact that Ψ_tot_ tends to be a better predictor of fitness than Ψ_del_ therefore implies that variants scored as nondeleterious by PROVEAN often have detectable effects in the lab. Indeed, if we examine models with both Ψ_del_ and Ψ_non__del_=Ψ_tot_−Ψ_del_ as predictors, we find that Ψ_non__del_ is a significant predictor of fitness (in all data sets). This implies that the effect of an allele in one environment (the lab) might overestimate its long-term effect. Although the conventional wisdom is that mutations might have weaker effects in a simple laboratory environment than they do in putatively stressful natural environments, there is little support for the idea that stress consistently increases the strength of selection (Agrawal and Whitlock 2010).

Despite PROVEAN calling large protein changes “deleterious,” mutations at phylogenetically conserved sites can have effects that are both positive and negative. Indeed, mutations scored as deleterious by PROVEAN have been found to be adaptive in stressful conditions (Gorter et al. 2017), and many mutations that have improved crops during plant domestication are scored as deleterious by the program (Kono et al. 2016). Furthermore, the potential for epistatic interactions among alleles means that fitness need not be the sum of its parts, that is, mutations may exacerbate or compensate for the effects of one another. This may be particularly relevant when using the presence of deleterious variants to infer high mutation load; in the presence of deleterious alleles, selection can favor the spread of other deleterious alleles due to positive epistasis (compensatory adaptation, e.g., Trindade et al. [2009]).

Whenever the effects of alleles depend on the environment or genetic background, we should expect a weakening of the association between PROVEAN scores and the realized impacts of mutations in a given context. By restricting the analysis to genes that are essential in the standard laboratory environment in *S. cerevisiae*, we might expect to find a stronger correlation between fitness and PROVEAN score, though the size of the data set is reduced. However, the number of mutant essential proteins was never preferred over the total number of mutant proteins in our model comparisons.

Since PROVEAN scores are based on patterns that manifest on an evolutionary timescale, it makes sense that they would have the greatest predictive value in terms of the long-term impacts of protein variants. If this framework is correct, it would imply that tools like PROVEAN are best used to obtain global, long-term predictions, but would have weaker predictive power in any single genetic or environmental context.

We should also recognize that the standing frequency of a deleterious allele depends not just on its fitness effect, but also the rate at which it is generated by mutation. In principle, alleles classified as neutral by PROVEAN due to their high frequency could instead have mildly deleterious effects and a high underlying mutation rate. The potential magnitude of this effect is unclear, but we do know that single-nucleotide mutations appearing in the Sc1 MA experiment coincide with polymorphic sites identified in global samples of yeast (Schacherer et al. 2009) more often than expected by chance (Sharp et al. 2018), indicating a detectable level of mutation rate variation across the genome that persists among genetic backgrounds.

Our models will also be less effective if there are mutations among the MA lines that were not detected at all. In the yeast data sets most of the genome was sequenced to moderate or high depth, and so it is unlikely that many mutations in protein-coding regions were missed. In the Cr data set, about 62.8% of the genome was successfully genotyped, so the risk of undetected mutations may be greater, but this is also the data set where PROVEAN was most successful. Although we attempted to account for the effects of structural variation in the yeast data sets, we did not consider mutations in mitochondrial or chloroplast genomes.

In the MA data sets we studied the ancestral protein sequences are known, but in many eco-evolutionary studies mutant sequences may instead be compared with reference sequences. The predicted fitness effect of nonsynonymous changes is dependent on the query sequence, something reported on first in human population genetic studies. Simons et al. (2014) counted the average number of derived alleles at putatively deleterious sites per individual in Americans of European or African ancestry to test the mutational load in the two populations in relation to their demographic history, using PolyPhen-2 to choose what segregating variants to consider as deleterious. They found a strong bias in the way the program scored the variants: if the reference genome carried the derived variant, it was more likely to be scored as benign than if the reference genome carried the ancestral version of the protein. To examine this effect in our study, we conducted an exploratory analysis of the PROVEAN score of variants differentiating the genomes of ancestral controls from their respective reference genomes. We consider this analysis to be closest to the situation in which it is employed in many eco-evolutionary studies (relative fitness is unknown, and comparisons are made to a reference genome). Our findings suggest that the laboratory ancestors used in MA experiments we examined carried deleterious alleles relative to the reference type, except when closely related to the reference strain, as in the case of Sc2; these alleles appear to be less deleterious than spontaneous mutations in *S. cerevisiae*, but more deleterious in *C. reinhardtii*. However, we only examined proteins in which new mutations arose during MA, whereas reference and ancestral strains may differ at many additional loci (Craig et al. 2019). The data underlying *C. reinhardtii* are also more limited: the mean E-value for supporting sequences is ten times lower (better) for yeast than for *C. reinhardtii*. This could partly be explained by yeast having shorter proteins on average (Sc1: 777 amino acids, Sc2: 767, Cr: 1,310). Shorter proteins will have higher E-values because homology is more likely to occur by chance. Still, there is a significant difference in the E-value when submitting protein sequences from the *C. reinhardtii* reference genome as compared with proteins of the field isolates used to initiate MA in the Cr experiment. This may reflect a bias in the sequences reported to NCBI such that sequences of commonly used strains and model organisms compose a large portion of the database, whereas less-studied taxa and natural isolates are underrepresented.

These findings indicate that caution should be used when applying PROVEAN to strains or species that are not common in the NCBI database. In principle, the use of highly divergent query sequences is not an issue because PROVEAN uses the difference in alignment score between query and mutant sequence rather than the absolute alignment score. However, variation shared by all individuals in a study (analogous to the differences between MA ancestors and the reference genome) can matter for PROVEAN scores, particularly in the case of indels. We recommend that researchers first incorporate any shared differences from the reference into query protein sequences before evaluating additional polymorphisms. For different kinds of reference bias and suggestions of how to address these we refer the reader to Moyers et al. (2018).

Importantly, PROVEAN and similar alignment-based tools are designed to annotate individual variants but are frequently applied to genotypes with multiple variants. The aggregate result of multiple variants on fitness is indirect and will depend on patterns of dominance and epistasis (Henn et al. 2015, 2016) that we cannot evaluate here. Although our results do indicate that counting the number of proteins with derived variants can serve as a proxy for fitness, at least in MA lines, using this approach to measure the relative health or mutation load of populations in the wild may be less effective, particularly if adaptive protein changes are common.

### Conclusion

Although bioinformatic tools that assign genetic variants to binary fitness effect categories may be attractive for their simplicity, care should be taken when applying these tools in contexts where they have not been tested and calibrated. We find that the information provided by PROVEAN has predictive value, but that better accuracy can often be obtained by simply considering the total number of variant proteins as a proxy for fitness change. However, we also find evidence that there can be value in the PROVEAN scores themselves, beyond a binary classification, based on some of the cases we studied. We encourage further studies evaluating the applicability of mutation scoring algorithms in eco-evolutionary settings.

## Materials and Methods

### Data Processing

#### *Saccharomyces cerevisiae* Data Set 1 (Sc1)

This experiment is described in detail in Sharp et al. (2018). In short, 220 replicate yeast lines were propagated under relaxed selection for up to 100 days (∼1,500 generations per line on average). The 220 lines were roughly equally distributed among four groups: haploids and diploids, with either their copy of the *RDH54* gene (responsible for homologous recombination and repair during mitosis) deleted or intact. At the end of the experiment, the growth rate of 218 of these lines and 182 replicates of their ancestors (equally distributed among the four groups), were measured in two Bioscreen C machines. The growth rate assay was done 11 times, over consecutive days. The growth rates from one machine on one day was omitted from the data analysis because of a shaking malfunction, such that the number of observations varies between 10 and 11 for each line. There was a reduction in the mean growth rate of diploid, but not haploid, MA lines compared with controls. Nevertheless, there was significant genetic variation in all groups of MA lines, but in no group of the control lines (evaluated by likelihood ratio test of mixed effect model with or without line identity as an explanatory factor; Sharp et al. 2018). The genomic analysis revealed dozens of cases of aneuploidy in the diploid lines, accounting for a large part, but not all, of the reduction in fitness in this treatment.

We used the mutations reported in the supplementary data of Sharp et al. (2018). There were 1,474 genic mutations in the data set, occurring in 1,219 unique genes across 218 MA lines. We extracted the nucleotide and protein sequence of the genes affected using YeastMine (Balakrishnan et al. 2012). From the same database, we downloaded the location of introns in these genes. The reference nucleotide sequence was then mutated in silico to represent the mutant sequence, which was then transcribed and translated, using the seqinr package (Charif and Lobry 2007) in R (R Core Team 2021). Additionally, we analyzed VCF files to obtain a table of mutations in the ancestral line as compared with the yeast reference genome (version R64-2-1). In cases where the ancestor and reference strain differed for a mutated gene (126 genes), we separately computed the ancestral protein and used it for comparison to the MA lines. We wrote a script to produce protein variants in the format PROVEAN requires. From 1,474 genic mutations, 1,126 protein variants were computed (in 961 unique proteins). Two samples (lines 113 and 206) had no nonsynonymous mutations. When an MA line had more than one nonsynonymous mutation in a particular gene both mutations were considered when altering the protein and the number of mutant proteins is reported once. Out of 961 altered proteins, 126 already differed between the S288C reference genome and the laboratory ancestor, in which case the latter was used as the query sequence.

The growth rates reported in the supplementary data of Sharp et al. (2018) include all replicate measurements for each line. We evaluated the percentage of variation in growth rate explained by line identity in the ancestor and MA lines. In the control group, where each line was an isogenic copy of the ancestor, 2% of variance could be attributable to line identity. In the MA lines, 15% of variance was attributable to differences among lines. The intraclass correlation for MA line was 0.169 (computed with the “performance” package [Lüdecke et al. 2021] in R [R Core Team 2021]). Unless otherwise noted, we used all replicate measurements of each MA line to create one response variable that we call relative fitness: the average growth rate of each MA line compared with the average growth rate of the ancestral line with the same mating type, ploidy (haploid or diploid), and *RDH54* gene, accounting for random block effects. There were two lines for which there was no growth rate data; these lacked mitochondrial function (petite) by the end of the MA experiment and were excluded from the analysis (lines 110 and 189).

#### *Saccharomyces cerevisiae* Data Set 2 (Sc2)

This experiment is described in detail in Liu and Zhang (2019). In short, 168 diploid yeast lines were propagated under relaxed selection for around 60 bottlenecks under continuous growth (∼1,000 generations per line). The 168 lines were equally distributed among seven treatment groups that were grown on different kinds of media. Two lines were lost during MA, likely due to lethal mutations. At the end of the experiment, the growth rate of frozen samples from the beginning of the experiment and evolved MA lines were measured through cell counts of single colonies (one colony per line) on the same medium as that on which they evolved. There was a general reduction in mean growth rate of the MA lines compared with controls. The genomic analysis revealed multiple aneuploidy events and segmental duplications in two environments, which could explain a large part, but not all, of the reduction in fitness.

We used the mutations reported in the supplementary data of Liu and Zhang (2019). Additionally, the authors supplied us with a table of mutations in their ancestral line relative to the S288C reference genome. We used the same method as described above for data set Sc1. There were 1,147 genic mutations, occurring in 968 unique genes, across 165 MA lines. From 1,147 genic mutations, 877 protein variants were computed (in 754 unique proteins). Out of 754 altered proteins, 16 already differed between the S288C reference genome and the laboratory ancestor, in which case the latter was used as the query sequence.

Growth rates in five different media environments were reported in the supplementary material of Liu and Zhang (2019). Because each line was measured only once, we cannot produce a repeatability estimate. However, we can see that the variance among measurements of the ancestors (23–24 measurements in each condition) is smaller than the variance among MA lines (*F* value = 28.23, *P* < 10^−5^). We computed the relative fitness of each evolved MA line by subtracting the mean initial growth rate of the starting strains in that focal medium from the final growth rate of each line in that medium.

In addition, we used the information on chromosome aneuploidies and segmental duplications and deletions from the supplementary table of Liu and Zhang (2019) to compute a relative genome size for each strain.

#### *Chlamydomonas reinhardtii* Data Set (Cr)

This experiment is described in detail in Morgan *et al.* (2014). In short, 15 replicate lines from each of six different *C. reinhardtii* strains (for a total of 90 MA lines) were propagated under relaxed selection for around 85 bottlenecks under continuous growth (∼1,000 generations per line). Following MA, the experimenters measured growth rates of replicates of the ancestor and MA lines and found an increase in variance in fitness as well as a reduction in mean fitness in the MA lines. The genomic analysis of these lines was presented in Ness et al. (2015). Competitive growth rates of the MA lines and ancestors were reported in Kraemer et al. (2017), where they found significant correlations between the number of nonsynonymous mutations in coding regions and the growth rates of MA lines.

We received an annotated table of the mutations reported in Ness et al. (2015) as well as VCF files containing the mutations in their six ancestral lines compared with the reference genome. We downloaded an annotated table for all transcripts in the *Chlamydomonas* reference genome from Dicots PLAZA 4.0 (version 5.5, Van Bel et al. 2018) to identify mutations in coding sequences. Out of the original 6,843 mutations, 3,889 affected protein sequence, representing 1,439 mutated proteins after combining mutations. We found that most transcripts that were mutated during MA already had existing variants in the ancestral strain, relative to the reference (table 1). About 1,397 out of the originally predicted 1,439 protein variants remained once ancestral variation had been considered (table 1). As in the other data sets, we use the ancestral protein as the query protein.

We found two cases in the *C. reinhardtii* data set where the reported reference nucleotide deviated from that found in the Dicots PLAZA 4.0 sequence; in each case, the differences between the two reference sequences were synonymous. This discrepancy was likely due to the two different reference genomes used (Ness et al. used v5.3; Van Bel et al. used v5.5).

To test the accuracy of our sequence-mutating code, we mutated the coding sequence to the reference nucleotide given by the *C. reinhardtii* data set and verified that this produced the reference transcript. We converted the proteins carrying variants into the format PROVEAN requires. In cases with alternative transcripts, we treat these as separate proteins in PROVEAN and then report the minimum score given to any protein variant of a gene. This occurred in 42 unique cases, involving all genetic backgrounds. Although the difference in scores between transcripts in general was small, we found two cases where the score for one affected transcript was below the default threshold of −2.5 whereas the other was above it, and six cases where the scores fell above and below zero. Six out of the total 1,397 proteins carrying variants failed to receive a score from PROVEAN, likely because the changes to the protein were too large to compute alignment scores between the clusters gathered and the mutant protein and were ignored in the analysis (these occurred in six different samples across five ancestral backgrounds).

Growth rates for each MA line were found in the DRYAD repository for Kraemer et al. (2017). MA lines were measured in a benign environment and in medium supplemented with 2.5 g/l of NaCl (referred to as moderate stress). We followed Kraemer et al. (2017) and excluded three lines for which they estimated extreme mutation rate values; 72 MA lines remained. Because the researchers did not find an effect of media treatment on competitive fitness of their lines (table 3 of Kraemer et al. [2017]), we used the estimated fitness in the benign environment for our analysis. Kraemer et al. (2017) report competitive fitness (calculated as the growth rate of the focal strain minus the growth rate of the competitor strain) for all replicate measurements for each MA line and ancestor. The percentage of variance in competitive fitness attributed to line identity in the MA lines was 39%. The intraclass correlation for line identity was 0.40 (computed with the performance package [Lüdecke et al. 2021] in R [R Core Team 2021]). We used all replicates to create one response variable that we refer to as relative competitive fitness: the average growth rate of each MA line compared with the average growth rate of its ancestor, accounting for the random block effect of plate.

### Running PROVEAN

We ran PROVEAN on the ComputeCanada cluster. As the program failed to run with the recent BLAST software (version 2.9.0), we configured PROVEAN to run with PSI-BLAST and BLASTDBCMD (Altschul et al. 1997) from BLAST version 2.4.0. We used version 4.8.1 of CD-HIT. We ran our variants with the NCBI nr database from November 11, 2019, which holds 142 GB of nonredundant sequences (229,636,095 sequences). We ran a subset of variants using the 2012 database, on which PROVEAN was developed (the first 5 GB), without radical changes to the PROVEAN scores of variants. The supporting sequence sets used to compute the alignment scores for all proteins were saved.

We supplied the protein of the ancestor as the query sequence. It should be noted that PROVEAN has not been validated for frameshift or nonsense mutations. We have several proteins carrying variants that cause large changes to the protein. These all received very negative scores (table 2). One variant protein also received a large positive score (+1,342, which PROVEAN calls neutral). This happened in line CC1373_7 in the *Cr* data set, where a frameshift that had caused a premature stop codon in the ancestral line CC1373 was reverted.

**Table 2:** Summary of Protein Mutations and Scores in Sc1, Sc2, and Cr Data Set

	Type	Number	Max Score	Min Score
Sc1	Single aa substitution	1,015	5.22	−14
	Duplication	5	0.57	−3.74
	Deletion	71	0.81	−4,000
	Insertion	2	1.64	−4.78
	Complex	37	−0.75	−2,650
Sc2	Single aa substitution	721	7.37	−14
	Duplication	24	2.08	−9.54
	Deletion	62	−1.48	−5,978
	Insertion	1	−0.17	−0.17
	Complex	61	4.07	−2,910
Cr data set	Single aa substitution	1,263	5.18	−12.7
	Duplication	27	1.5	−6.84
	Deletion	74	0.58	−4,259
	Insertion	19	1.83	−8
	Complex	141	1,343	−6,620

Note.—Complex mutations refer to cases where more than one kind of mutation (substitution, insertion, or deletion) occur in the same protein.

### Statistical Analyses

We fit linear models of relative fitness with a fixed intercept at zero (representing the fitness of the control lines that did not carry mutations). In our analysis, we use several summary statistics. The first is the number of mutant proteins per line, Ψ_tot_. This simply reflects the number of altered proteins in a line regardless of their PROVEAN scores. The second summary statistic is the number of mutant proteins classified as deleterious using the default cutoff of −2.5 by PROVEAN, Ψ_del_. Because some studies have used the absolute departure from zero (Yoshida et al. 2016, 2019; Kusakabe et al. 2017), we also investigate the number of proteins with variants scored below −2.5 or above 2.5, Ψ_abs_. We also investigated whether there is any quantitative information in the PROVEAN score itself, beyond the categorical information (neutral or deleterious), by fitting models using the sum of PROVEAN scores. However, since very misaligned sequences receive extreme scores leading to a skewed distribution, we decided to log-transform the values of the scores: ∑_tot_=log(1−sum of scores).

In an attempt to account for the importance of a protein to fitness, we used a previously published list of essential genes in yeast (Winzeler et al. 1999, downloaded from the Yeast Deletion Web Pages, http://www-sequence.stanford.edu/group/yeast_deletion_project/deletions3.html, last accessed June 2019) and added the “essentialness” of a protein as an explanatory factor in our models of the yeast data sets. Summary variables by the essentialness of the proteins are labeled Ψ_tot_ess_, Ψ_del_ess_, Ψ_abs_ess_, and ∑_tot_ess_. We compared models using the R package AICcmodavg (Mazerolle 2020). We used this package to produce model selection tables based on second order AIC.

#### *Saccharomyces cerevisiae* Data Set 1

We analyzed haploids and diploids separately, given that mutational effects may differ between them due to masking in diploids, using linear models. In the diploids, relative genome size was included as a predictor variable. Because haploid MA lines did not experience an overall decrease in growth rate (but still showed genetic variance in fitness), we used models with the absolute value of relative fitness (a decrease and increase in fitness of the same magnitude will count the same). 18% of the proteins with variants occurred in essential genes (197 out of 1,130).

#### *Saccharomyces cerevisiae* Data Set 2

We used linear models of relative fitness as a function of each predictor. We combined our analysis of the strains from the different environments as we found no significant effect of environment on the effect of MA (*F* = 1.38, *P* = 0.22). Similarly, we did not find a significant effect on fitness of relative genome size (*P* = 0.766, computed by adding or subtracting potentially lost or gained chromosomes or large segmental deletions or insertions), and chose to ignore these large-scale genomic changes in our analysis. 20% of the proteins with variants occurred in essential genes (178 out of 877).

#### *Chlamydomonas reinhardtii* Data Set

We used linear models of relative fitness as a function of each PROVEAN predictor and included ancestral background as a random effect on the slope. Because competitive fitness only decreased significantly after MA in two out of six ancestral backgrounds (CC2342 and CC2931), our models use the absolute value of competitive fitness as the response variable. 

## Supplementary Material

Supplementary data are available at *Genome Biology and Evolution* online.

## Supplementary Material

evac004_Supplementary_Data
