# Supplementary material for: Fitness Effects of Mutations: An Assessment of PROVEAN Predictions Using Mutation Accumulation Data
Source: Genome Biol Evol. 2022 Jan 17;14(1):evac004. doi: 10.1093/gbe/evac004 (PMC8790079; doi:10.1093/gbe/evac004)
Supplement: evac004_Supplementary_Data [file evac004_supplementary_data.zip › SupplementaryModels.pdf]

Supplementary material for PROVEAN project

Linnea Sandell

06/11/2021

Models in Sc1 haploids

All haploid models were run with the absolute change in growth rate as the response variable. We first evaluate models with a fixed intercept at 0, and then models without this fixed intercept.

Models with fixed intercept at 0.

```
##
## Call:
## lm(formula = abs(rel.w) ~ 0 + tot, data = sc1[sc1$ploidy == "hap",
##     ])
##
## Residuals:
##       Min        1Q    Median        3Q       Max
## -0.004725 -0.001372  0.000633  0.001621  0.009702
##
## Coefficients:
##      Estimate Std. Error t value Pr(>|t|)
## tot 5.751e-04  5.631e-05   10.21   <2e-16 ***
## ---
## Signif. codes:  0 '***' 0.001 '**' 0.01 '*' 0.05 '.' 0.1 ' ' 1
##
## Residual standard error: 0.002698 on 103 degrees of freedom
##   (2 observations deleted due to missingness)
## Multiple R-squared:  0.5031, Adjusted R-squared:  0.4983
## F-statistic: 104.3 on 1 and 103 DF,  p-value: < 2.2e-16
```

```
##
## Call:
## lm(formula = abs(rel.w) ~ 0 + del, data = sc1[sc1$ploidy == "hap",
##     ])
##
## Residuals:
##        Min         1Q     Median         3Q        Max
## -0.0047651 -0.0009445  0.0008620  0.0020570  0.0098560
##
## Coefficients:
##      Estimate Std. Error t value Pr(>|t|)
## del 0.0009964  0.0001075   9.268 3.14e-15 ***
## ---
```

```
## Signif. codes:  0 '***' 0.001 '**' 0.01 '*' 0.05 '.' 0.1 ' ' 1
##
## Residual standard error: 0.002826 on 103 degrees of freedom
##   (2 observations deleted due to missingness)
## Multiple R-squared:  0.4547, Adjusted R-squared:  0.4494
## F-statistic: 85.89 on 1 and 103 DF,  p-value: 3.139e-15
```

```
##
## Call:
## lm(formula = abs(rel.w) ~ 0 + score_sum, data = sc1[sc1$ploidy ==
##     "hap", ])
##
## Residuals:
##        Min         1Q     Median         3Q        Max
## -0.0046524 -0.0007135  0.0008328  0.0023562  0.0102950
##
## Coefficients:
##             Estimate Std. Error t value Pr(>|t|)
## score_sum -1.426e-04  1.509e-05  -9.449 1.24e-15 ***
## ---
## Signif. codes:  0 '***' 0.001 '**' 0.01 '*' 0.05 '.' 0.1 ' ' 1
##
## Residual standard error: 0.002801 on 103 degrees of freedom
##   (2 observations deleted due to missingness)
## Multiple R-squared:  0.4643, Adjusted R-squared:  0.4591
## F-statistic: 89.29 on 1 and 103 DF,  p-value: 1.242e-15
```

```
##
## Call:
## lm(formula = abs(rel.w) ~ 0 + tot_ess, data = sc1[sc1$ploidy ==
##     "hap", ])
##
## Residuals:
##        Min         1Q     Median         3Q        Max
## -0.0046341  0.0002211  0.0016154  0.0029652  0.0108524
##
## Coefficients:
##          Estimate Std. Error t value Pr(>|t|)
## tot_ess 0.0018279  0.0003232   5.656  1.4e-07 ***
## ---
## Signif. codes:  0 '***' 0.001 '**' 0.01 '*' 0.05 '.' 0.1 ' ' 1
##
## Residual standard error: 0.003343 on 103 degrees of freedom
##   (2 observations deleted due to missingness)
## Multiple R-squared:  0.237,  Adjusted R-squared:  0.2296
## F-statistic: 31.99 on 1 and 103 DF,  p-value: 1.397e-07
```

```
##
## Call:
## lm(formula = abs(rel.w) ~ 0 + del_ess, data = sc1[sc1$ploidy ==
##     "hap", ])
##
## Residuals:
```

```
##        Min         1Q     Median         3Q        Max
## -0.0037411  0.0006005  0.0019219  0.0036679  0.0116293
##
## Coefficients:
##          Estimate Std. Error t value Pr(>|t|)
## del_ess 0.0015303  0.0004887   3.131  0.00227 **
## ---
## Signif. codes:  0 '***' 0.001 '**' 0.01 '*' 0.05 '.' 0.1 ' ' 1
##
## Residual standard error: 0.003657 on 103 degrees of freedom
##   (2 observations deleted due to missingness)
## Multiple R-squared:  0.08692,    Adjusted R-squared:  0.07806
## F-statistic: 9.805 on 1 and 103 DF,  p-value: 0.002265
```

```
##
## Call:
## lm(formula = abs(rel.w) ~ 0 + sum_ess, data = sc1[sc1$ploidy ==
##     "hap", ])
##
## Residuals:
##        Min         1Q     Median         3Q        Max
## -0.0050518  0.0005617  0.0018840  0.0036679  0.0117119
##
## Coefficients:
##           Estimate Std. Error t value Pr(>|t|)
## sum_ess -0.0002857  0.0000815  -3.506 0.000676 ***
## ---
## Signif. codes:  0 '***' 0.001 '**' 0.01 '*' 0.05 '.' 0.1 ' ' 1
##
## Residual standard error: 0.003617 on 103 degrees of freedom
##   (2 observations deleted due to missingness)
## Multiple R-squared:  0.1066, Adjusted R-squared:  0.09792
## F-statistic: 12.29 on 1 and 103 DF,  p-value: 0.0006762
```

```
##
## Call:
## lm(formula = abs(rel.w) ~ 0 + nondel, data = sc1[sc1$ploidy ==
##     "hap", ])
##
## Residuals:
##        Min         1Q     Median         3Q        Max
## -0.0046291 -0.0008070  0.0007412  0.0020520  0.0098139
##
## Coefficients:
##         Estimate Std. Error t value Pr(>|t|)
## nondel 0.0010384  0.0001167   8.898 2.08e-14 ***
## ---
## Signif. codes:  0 '***' 0.001 '**' 0.01 '*' 0.05 '.' 0.1 ' ' 1
##
## Residual standard error: 0.002878 on 103 degrees of freedom
##   (2 observations deleted due to missingness)
## Multiple R-squared:  0.4346, Adjusted R-squared:  0.4291
## F-statistic: 79.17 on 1 and 103 DF,  p-value: 2.076e-14
```

```
##
## Call:
## lm(formula = abs(rel.w) ~ 0 + nondel_ess, data = sc1[sc1$ploidy ==
##     "hap", ])
##
## Residuals:
##        Min         1Q     Median         3Q        Max
## -0.0038948  0.0004528  0.0018649  0.0037325  0.0108524
##
## Coefficients:
##            Estimate Std. Error t value Pr(>|t|)
## nondel_ess 0.002970   0.000557   5.333 5.76e-07 ***
## ---
## Signif. codes:  0 '***' 0.001 '**' 0.01 '*' 0.05 '.' 0.1 ' ' 1
##
## Residual standard error: 0.003388 on 103 degrees of freedom
##   (2 observations deleted due to missingness)
## Multiple R-squared:  0.2163, Adjusted R-squared:  0.2087
## F-statistic: 28.44 on 1 and 103 DF,  p-value: 5.76e-07
```

Models without fixed intercept

```
##
## Call:
## lm(formula = abs(rel.w) ~ tot, data = sc1[sc1$ploidy == "hap",
##     ])
##
## Residuals:
##        Min         1Q     Median         3Q        Max
## -0.0031730 -0.0017593 -0.0005116  0.0011902  0.0087251
##
## Coefficients:
##              Estimate Std. Error t value Pr(>|t|)
## (Intercept) 0.0023604  0.0004946   4.772  6.1e-06 ***
## tot         0.0001359  0.0001053   1.291      0.2
## ---
## Signif. codes:  0 '***' 0.001 '**' 0.01 '*' 0.05 '.' 0.1 ' ' 1
##
## Residual standard error: 0.002451 on 102 degrees of freedom
##   (2 observations deleted due to missingness)
## Multiple R-squared:  0.01608,    Adjusted R-squared:  0.006431
## F-statistic: 1.667 on 1 and 102 DF,  p-value: 0.1996
```

```
##
## Call:
## lm(formula = abs(rel.w) ~ del, data = sc1[sc1$ploidy == "hap",
##     ])
##
## Residuals:
##        Min         1Q     Median         3Q        Max
## -0.0031074 -0.0016857 -0.0005608  0.0011523  0.0087335
##
## Coefficients:
```

```
##              Estimate Std. Error t value Pr(>|t|)
## (Intercept) 0.0024665  0.0004165   5.921 4.34e-08 ***
## del         0.0002147  0.0001616   1.328    0.187
## ---
## Signif. codes:  0 '***' 0.001 '**' 0.01 '*' 0.05 '.' 0.1 ' ' 1
##
## Residual standard error: 0.00245 on 102 degrees of freedom
##   (2 observations deleted due to missingness)
## Multiple R-squared:  0.01701,    Adjusted R-squared:  0.007371
## F-statistic: 1.765 on 1 and 102 DF,  p-value: 0.187

##
## Call:
## lm(formula = abs(rel.w) ~ score_sum, data = sc1[sc1$ploidy ==
##     "hap", ])
##
## Residuals:
##        Min         1Q     Median         3Q        Max
## -0.0031059 -0.0017578 -0.0004387  0.0011873  0.0083954
##
## Coefficients:
##               Estimate Std. Error t value Pr(>|t|)
## (Intercept)  2.295e-03  3.827e-04   5.996 3.09e-08 ***
## score_sum   -4.367e-05  2.102e-05  -2.077   0.0403 *
## ---
## Signif. codes:  0 '***' 0.001 '**' 0.01 '*' 0.05 '.' 0.1 ' ' 1
##
## Residual standard error: 0.00242 on 102 degrees of freedom
##   (2 observations deleted due to missingness)
## Multiple R-squared:  0.04059,    Adjusted R-squared:  0.03118
## F-statistic: 4.315 on 1 and 102 DF,  p-value: 0.04029

##
## Call:
## lm(formula = abs(rel.w) ~ tot_ess, data = sc1[sc1$ploidy == "hap",
##     ])
##
## Residuals:
##        Min         1Q     Median         3Q        Max
## -0.0029487 -0.0017684 -0.0006249  0.0011576  0.0085889
##
## Coefficients:
##              Estimate Std. Error t value Pr(>|t|)
## (Intercept) 2.863e-03  3.075e-04   9.311 2.73e-15 ***
## tot_ess     8.862e-05  3.032e-04   0.292    0.771
## ---
## Signif. codes:  0 '***' 0.001 '**' 0.01 '*' 0.05 '.' 0.1 ' ' 1
##
## Residual standard error: 0.00247 on 102 degrees of freedom
##   (2 observations deleted due to missingness)
## Multiple R-squared:  0.000837,   Adjusted R-squared:  -0.008959
## F-statistic: 0.08545 on 1 and 102 DF,  p-value: 0.7706

##
```

```
## Call:
## lm(formula = abs(rel.w) ~ del_ess, data = sc1[sc1$ploidy == "hap",
##     ])
##
## Residuals:
##        Min         1Q     Median         3Q        Max
## -0.0030232 -0.0017435 -0.0006134  0.0012103  0.0085567
##
## Coefficients:
##               Estimate Std. Error t value Pr(>|t|)
## (Intercept)  0.0030726  0.0002729  11.259   <2e-16 ***
## del_ess     -0.0004450  0.0003719  -1.197    0.234
## ---
## Signif. codes:  0 '***' 0.001 '**' 0.01 '*' 0.05 '.' 0.1 ' ' 1
##
## Residual standard error: 0.002454 on 102 degrees of freedom
##   (2 observations deleted due to missingness)
## Multiple R-squared:  0.01384,    Adjusted R-squared:  0.004173
## F-statistic: 1.432 on 1 and 102 DF,  p-value: 0.2343

##
## Call:
## lm(formula = abs(rel.w) ~ sum_ess, data = sc1[sc1$ploidy == "hap",
##     ])
##
## Residuals:
##        Min         1Q     Median         3Q        Max
## -0.0029053 -0.0017934 -0.0006266  0.0011120  0.0086962
##
## Coefficients:
##              Estimate Std. Error t value Pr(>|t|)
## (Intercept) 2.931e-03  2.690e-04  10.898   <2e-16 ***
## sum_ess     6.690e-06  6.180e-05   0.108    0.914
## ---
## Signif. codes:  0 '***' 0.001 '**' 0.01 '*' 0.05 '.' 0.1 ' ' 1
##
## Residual standard error: 0.002471 on 102 degrees of freedom
##   (2 observations deleted due to missingness)
## Multiple R-squared:  0.0001149,  Adjusted R-squared:  -0.009688
## F-statistic: 0.01172 on 1 and 102 DF,  p-value: 0.914

##
## Call:
## lm(formula = abs(rel.w) ~ nondel, data = sc1[sc1$ploidy == "hap",
##     ])
##
## Residuals:
##        Min         1Q     Median         3Q        Max
## -0.0030421 -0.0017529 -0.0005617  0.0011678  0.0087108
##
## Coefficients:
##              Estimate Std. Error t value Pr(>|t|)
## (Intercept) 0.0026653  0.0004301   6.197 1.23e-08 ***
## nondel      0.0001266  0.0001779   0.712    0.478
```

```
## ---
## Signif. codes:  0 '***' 0.001 '**' 0.01 '*' 0.05 '.' 0.1 ' ' 1
##
## Residual standard error: 0.002465 on 102 degrees of freedom
##   (2 observations deleted due to missingness)
## Multiple R-squared:  0.004944,   Adjusted R-squared:  -0.004812
## F-statistic: 0.5068 on 1 and 102 DF,  p-value: 0.4782
```

```
##
## Call:
## lm(formula = abs(rel.w) ~ nondel_ess, data = sc1[sc1$ploidy ==
##     "hap", ])
##
## Residuals:
##        Min         1Q     Median         3Q        Max
## -0.0032466 -0.0018185 -0.0005696  0.0011887  0.0081774
##
## Coefficients:
##             Estimate Std. Error t value Pr(>|t|)
## (Intercept) 0.0026750  0.0002692   9.937   <2e-16 ***
## nondel_ess  0.0008735  0.0004513   1.935   0.0557 .
## ---
## Signif. codes:  0 '***' 0.001 '**' 0.01 '*' 0.05 '.' 0.1 ' ' 1
##
## Residual standard error: 0.002427 on 102 degrees of freedom
##   (2 observations deleted due to missingness)
## Multiple R-squared:  0.03542,    Adjusted R-squared:  0.02596
## F-statistic: 3.746 on 1 and 102 DF,  p-value: 0.05571
```

AIC of haploid models

```
##
## Model selection based on AIC:
##
##                K     AIC Delta_AIC AICWt Cum.Wt     LL
## sum            3 -953.84      0.00  0.34   0.34 479.92
## n_nondel_ess   3 -953.28      0.56  0.26   0.60 479.64
## n_del          3 -951.32      2.53  0.10   0.69 478.66
## n_tot          3 -951.22      2.62  0.09   0.79 478.61
## n_del_ess      3 -950.98      2.86  0.08   0.87 478.49
## n_nondel       3 -950.05      3.79  0.05   0.92 478.02
## n_tot_ess      3 -949.62      4.22  0.04   0.96 477.81
## sum_ess        3 -949.54      4.30  0.04   1.00 477.77
## 0_n_tot        2 -932.26     21.58  0.00   1.00 468.13
## 0_sum          2 -924.44     29.40  0.00   1.00 464.22
## 0_n_del        2 -922.59     31.25  0.00   1.00 463.29
## 0_n_nondel     2 -918.82     35.02  0.00   1.00 461.41
## 0_n_tot_ess    2 -887.65     66.19  0.00   1.00 445.82
## 0_n_nondel_ess 2 -884.87     68.97  0.00   1.00 444.44
## 0_sum_ess      2 -871.24     82.60  0.00   1.00 437.62
## 0_n_del_ess    2 -868.97     84.87  0.00   1.00 436.49
```

Models in Sc1 diploids

Models with intercept fixed at 0.

```
##
## Call:
## lm(formula = rel.w ~ 0 + tot + gen.size.rel, data = sc1[sc1$ploidy ==
##     "dip", ])
##
## Residuals:
##        Min         1Q     Median         3Q        Max
## -0.0212188 -0.0037362 -0.0004922  0.0029749  0.0108962
##
## Coefficients:
##                Estimate Std. Error t value Pr(>|t|)
## tot          -6.510e-04  7.764e-05  -8.385 1.69e-13 ***
## gen.size.rel -2.261e-01  2.184e-02 -10.353  < 2e-16 ***
## ---
## Signif. codes:  0 '***' 0.001 '**' 0.01 '*' 0.05 '.' 0.1 ' ' 1
##
## Residual standard error: 0.005268 on 112 degrees of freedom
## Multiple R-squared:  0.7006, Adjusted R-squared:  0.6952
## F-statistic:   131 on 2 and 112 DF,  p-value: < 2.2e-16
```

```
##
## Call:
## lm(formula = rel.w ~ 0 + del + gen.size.rel, data = sc1[sc1$ploidy ==
##     "dip", ])
##
## Residuals:
##        Min         1Q     Median         3Q        Max
## -0.0231718 -0.0041182 -0.0008525  0.0027455  0.0088821
##
## Coefficients:
##                Estimate Std. Error t value Pr(>|t|)
## del          -0.0011369  0.0001446  -7.864 2.52e-12 ***
## gen.size.rel -0.2320524  0.0222146 -10.446  < 2e-16 ***
## ---
## Signif. codes:  0 '***' 0.001 '**' 0.01 '*' 0.05 '.' 0.1 ' ' 1
##
## Residual standard error: 0.005394 on 112 degrees of freedom
## Multiple R-squared:  0.686,  Adjusted R-squared:  0.6804
## F-statistic: 122.3 on 2 and 112 DF,  p-value: < 2.2e-16
```

```
##
## Call:
## lm(formula = rel.w ~ 0 + abs + gen.size.rel, data = sc1[sc1$ploidy ==
##     "dip", ])
##
## Residuals:
##        Min         1Q     Median         3Q        Max
## -0.0231718 -0.0041866 -0.0009392  0.0026787  0.0087743
##
```

```
## Coefficients:
##               Estimate Std. Error t value Pr(>|t|)
## abs          -0.0011121  0.0001437  -7.737 4.85e-12 ***
## gen.size.rel -0.2330746  0.0223249 -10.440  < 2e-16 ***
## ---
## Signif. codes:  0 '***' 0.001 '**' 0.01 '*' 0.05 '.' 0.1 ' ' 1
##
## Residual standard error: 0.005426 on 112 degrees of freedom
## Multiple R-squared:  0.6823, Adjusted R-squared:  0.6767
## F-statistic: 120.3 on 2 and 112 DF,  p-value: < 2.2e-16
```

```
##
## Call:
## lm(formula = rel.w ~ 0 + score_sum + gen.size.rel, data = sc1[sc1$ploidy ==
##     "dip", ])
##
## Residuals:
##       Min        1Q    Median        3Q       Max
## -0.022611 -0.003775 -0.001394  0.002654  0.009811
##
## Coefficients:
##               Estimate Std. Error t value Pr(>|t|)
## score_sum     1.482e-04  1.872e-05    7.92 1.89e-12 ***
## gen.size.rel -2.312e-01  2.218e-02  -10.43  < 2e-16 ***
## ---
## Signif. codes:  0 '***' 0.001 '**' 0.01 '*' 0.05 '.' 0.1 ' ' 1
##
## Residual standard error: 0.005381 on 112 degrees of freedom
## Multiple R-squared:  0.6876, Adjusted R-squared:  0.682
## F-statistic: 123.2 on 2 and 112 DF,  p-value: < 2.2e-16
```

```
##
## Call:
## lm(formula = rel.w ~ 0 + tot_ess + gen.size.rel, data = sc1[sc1$ploidy ==
##     "dip", ])
##
## Residuals:
##       Min        1Q    Median        3Q       Max
## -0.023172 -0.005243 -0.001653  0.002095  0.008733
##
## Coefficients:
##               Estimate Std. Error t value Pr(>|t|)
## tot_ess      -0.0024164  0.0003593  -6.726 7.72e-10 ***
## gen.size.rel -0.2506882  0.0228307 -10.980  < 2e-16 ***
## ---
## Signif. codes:  0 '***' 0.001 '**' 0.01 '*' 0.05 '.' 0.1 ' ' 1
##
## Residual standard error: 0.005672 on 112 degrees of freedom
## Multiple R-squared:  0.6528, Adjusted R-squared:  0.6466
## F-statistic: 105.3 on 2 and 112 DF,  p-value: < 2.2e-16
```

```
##
## Call:
```

```
## lm(formula = rel.w ~ 0 + del_ess + gen.size.rel, data = sc1[sc1$ploidy ==
##     "dip", ])
##
## Residuals:
##        Min         1Q     Median         3Q        Max
## -0.0231718 -0.0056991 -0.0025580  0.0008879  0.0099662
##
## Coefficients:
##               Estimate Std. Error t value Pr(>|t|)
## del_ess      -0.0036552  0.0006158  -5.936 3.34e-08 ***
## gen.size.rel -0.2468721  0.0238859 -10.335  < 2e-16 ***
## ---
## Signif. codes:  0 '***' 0.001 '**' 0.01 '*' 0.05 '.' 0.1 ' ' 1
##
## Residual standard error: 0.005862 on 112 degrees of freedom
## Multiple R-squared:  0.6292, Adjusted R-squared:  0.6226
## F-statistic: 95.03 on 2 and 112 DF,  p-value: < 2.2e-16
```

```
##
## Call:
## lm(formula = rel.w ~ 0 + abs_ess + gen.size.rel, data = sc1[sc1$ploidy ==
##     "dip", ])
##
## Residuals:
##        Min         1Q     Median         3Q        Max
## -0.0231718 -0.0057164 -0.0026890  0.0008746  0.0094515
##
## Coefficients:
##               Estimate Std. Error t value Pr(>|t|)
## abs_ess      -0.0033979  0.0005997  -5.666 1.15e-07 ***
## gen.size.rel -0.2496127  0.0240788 -10.366  < 2e-16 ***
## ---
## Signif. codes:  0 '***' 0.001 '**' 0.01 '*' 0.05 '.' 0.1 ' ' 1
##
## Residual standard error: 0.005925 on 112 degrees of freedom
## Multiple R-squared:  0.6212, Adjusted R-squared:  0.6144
## F-statistic: 91.82 on 2 and 112 DF,  p-value: < 2.2e-16
```

```
##
## Call:
## lm(formula = rel.w ~ 0 + sum_ess + gen.size.rel, data = sc1[sc1$ploidy ==
##     "dip", ])
##
## Residuals:
##        Min         1Q     Median         3Q        Max
## -0.0231718 -0.0057567 -0.0026160  0.0002849  0.0094561
##
## Coefficients:
##               Estimate Std. Error t value Pr(>|t|)
## sum_ess       4.068e-04  7.581e-05   5.366 4.39e-07 ***
## gen.size.rel -2.551e-01  2.417e-02 -10.555  < 2e-16 ***
## ---
## Signif. codes:  0 '***' 0.001 '**' 0.01 '*' 0.05 '.' 0.1 ' ' 1
##
```

```
## Residual standard error: 0.005994 on 112 degrees of freedom
## Multiple R-squared:  0.6123, Adjusted R-squared:  0.6053
## F-statistic: 88.42 on 2 and 112 DF,  p-value: < 2.2e-16
```

```
##
## Call:
## lm(formula = rel.w ~ 0 + nondel + gen.size.rel, data = sc1[sc1$ploidy ==
##     "dip", ])
##
## Residuals:
##        Min         1Q     Median         3Q        Max
## -0.0195680 -0.0042143 -0.0009837  0.0024769  0.0115395
##
## Coefficients:
##                Estimate Std. Error t value Pr(>|t|)
## nondel       -0.0012013  0.0001597  -7.524 1.43e-11 ***
## gen.size.rel -0.2323590  0.0226073 -10.278  < 2e-16 ***
## ---
## Signif. codes:  0 '***' 0.001 '**' 0.01 '*' 0.05 '.' 0.1 ' ' 1
##
## Residual standard error: 0.005478 on 112 degrees of freedom
## Multiple R-squared:  0.6762, Adjusted R-squared:  0.6704
## F-statistic:   117 on 2 and 112 DF,  p-value: < 2.2e-16
```

```
##
## Call:
## lm(formula = rel.w ~ 0 + nondel_ess + gen.size.rel, data = sc1[sc1$ploidy ==
##     "dip", ])
##
## Residuals:
##       Min        1Q    Median        3Q       Max
## -0.023172 -0.006064 -0.002410  0.001027  0.008307
##
## Coefficients:
##                Estimate Std. Error t value Pr(>|t|)
## nondel_ess   -0.0030798  0.0006546  -4.705 7.31e-06 ***
## gen.size.rel -0.2738458  0.0241972 -11.317  < 2e-16 ***
## ---
## Signif. codes:  0 '***' 0.001 '**' 0.01 '*' 0.05 '.' 0.1 ' ' 1
##
## Residual standard error: 0.006141 on 112 degrees of freedom
## Multiple R-squared:  0.593,  Adjusted R-squared:  0.5857
## F-statistic:  81.6 on 2 and 112 DF,  p-value: < 2.2e-16
```

Models without intercept fixed at 0.

```
##
## Call:
## lm(formula = rel.w ~ tot + gen.size.rel, data = sc1[sc1$ploidy ==
##     "dip", ])
##
## Residuals:
##        Min         1Q     Median         3Q        Max
```

```
## -0.0184519 -0.0027103  0.0005561  0.0031983  0.0099991
##
## Coefficients:
##                 Estimate Std. Error t value Pr(>|t|)
## (Intercept)  -4.767e-03  1.266e-03  -3.764 0.000269 ***
## tot           1.577e-05  1.917e-04   0.082 0.934617
## gen.size.rel -2.329e-01  2.073e-02 -11.234  < 2e-16 ***
## ---
## Signif. codes:  0 '***' 0.001 '**' 0.01 '*' 0.05 '.' 0.1 ' ' 1
##
## Residual standard error: 0.004983 on 111 degrees of freedom
## Multiple R-squared:  0.542,  Adjusted R-squared:  0.5338
## F-statistic: 65.69 on 2 and 111 DF,  p-value: < 2.2e-16
```

```
##
## Call:
## lm(formula = rel.w ~ del + gen.size.rel, data = sc1[sc1$ploidy ==
##     "dip", ])
##
## Residuals:
##        Min         1Q     Median         3Q        Max
## -0.0188305 -0.0026242  0.0004873  0.0030790  0.0098763
##
## Coefficients:
##                 Estimate Std. Error t value Pr(>|t|)
## (Intercept)  -0.0043413  0.0009601  -4.522 1.54e-05 ***
## del          -0.0001052  0.0002643  -0.398    0.692
## gen.size.rel -0.2313630  0.0205061 -11.283  < 2e-16 ***
## ---
## Signif. codes:  0 '***' 0.001 '**' 0.01 '*' 0.05 '.' 0.1 ' ' 1
##
## Residual standard error: 0.004979 on 111 degrees of freedom
## Multiple R-squared:  0.5427, Adjusted R-squared:  0.5344
## F-statistic: 65.86 on 2 and 111 DF,  p-value: < 2.2e-16
```

```
##
## Call:
## lm(formula = rel.w ~ abs + gen.size.rel, data = sc1[sc1$ploidy ==
##     "dip", ])
##
## Residuals:
##        Min         1Q     Median         3Q        Max
## -0.0186413 -0.0026661  0.0004487  0.0031355  0.0099371
##
## Coefficients:
##                 Estimate Std. Error t value Pr(>|t|)
## (Intercept)  -4.531e-03  9.700e-04  -4.671 8.47e-06 ***
## abs          -4.415e-05  2.640e-04  -0.167    0.867
## gen.size.rel -2.321e-01  2.050e-02 -11.320  < 2e-16 ***
## ---
## Signif. codes:  0 '***' 0.001 '**' 0.01 '*' 0.05 '.' 0.1 ' ' 1
##
## Residual standard error: 0.004982 on 111 degrees of freedom
## Multiple R-squared:  0.5421, Adjusted R-squared:  0.5339
```

```
## F-statistic: 65.71 on 2 and 111 DF,  p-value: < 2.2e-16
```

```
##
## Call:
## lm(formula = rel.w ~ score_sum + gen.size.rel, data = sc1[sc1$ploidy ==
##     "dip", ])
##
## Residuals:
##        Min         1Q     Median         3Q        Max
## -0.0189191 -0.0026537  0.0004895  0.0032092  0.0098011
##
## Coefficients:
##               Estimate Std. Error t value Pr(>|t|)
## (Intercept)  -4.174e-03  9.319e-04  -4.479 1.83e-05 ***
## score_sum     2.079e-05  3.330e-05   0.624    0.534
## gen.size.rel -2.306e-01  2.050e-02 -11.246  < 2e-16 ***
## ---
## Signif. codes:  0 '***' 0.001 '**' 0.01 '*' 0.05 '.' 0.1 ' ' 1
##
## Residual standard error: 0.004974 on 111 degrees of freedom
## Multiple R-squared:  0.5436, Adjusted R-squared:  0.5354
## F-statistic: 66.11 on 2 and 111 DF,  p-value: < 2.2e-16
```

```
##
## Call:
## lm(formula = rel.w ~ tot_ess + gen.size.rel, data = sc1[sc1$ploidy ==
##     "dip", ])
##
## Residuals:
##        Min         1Q     Median         3Q        Max
## -0.0189140 -0.0023560  0.0005234  0.0031616  0.0099395
##
## Coefficients:
##               Estimate Std. Error t value Pr(>|t|)
## (Intercept)  -0.0042578  0.0007206  -5.909 3.85e-08 ***
## tot_ess      -0.0003628  0.0004689  -0.774    0.441
## gen.size.rel -0.2320147  0.0202505 -11.457  < 2e-16 ***
## ---
## Signif. codes:  0 '***' 0.001 '**' 0.01 '*' 0.05 '.' 0.1 ' ' 1
##
## Residual standard error: 0.00497 on 111 degrees of freedom
## Multiple R-squared:  0.5445, Adjusted R-squared:  0.5363
## F-statistic: 66.34 on 2 and 111 DF,  p-value: < 2.2e-16
```

```
##
## Call:
## lm(formula = rel.w ~ del_ess + gen.size.rel, data = sc1[sc1$ploidy ==
##     "dip", ])
##
## Residuals:
##        Min         1Q     Median         3Q        Max
## -0.0190107 -0.0023866  0.0003876  0.0030587  0.0095083
##
```

```
## Coefficients:
##                Estimate Std. Error t value Pr(>|t|)
## (Intercept)  -0.0041612  0.0006098  -6.824  4.9e-10 ***
## del_ess      -0.0008954  0.0006581  -1.361    0.176
## gen.size.rel -0.2288514  0.0203103 -11.268  < 2e-16 ***
## ---
## Signif. codes:  0 '***' 0.001 '**' 0.01 '*' 0.05 '.' 0.1 ' ' 1
##
## Residual standard error: 0.004942 on 111 degrees of freedom
## Multiple R-squared:  0.5495, Adjusted R-squared:  0.5414
## F-statistic:  67.7 on 2 and 111 DF,  p-value: < 2.2e-16
```

```
##
## Call:
## lm(formula = rel.w ~ abs_ess + gen.size.rel, data = sc1[sc1$ploidy ==
##     "dip", ])
##
## Residuals:
##        Min         1Q     Median         3Q        Max
## -0.0188696 -0.0024418  0.0004351  0.0032493  0.0096372
##
## Coefficients:
##                Estimate Std. Error t value Pr(>|t|)
## (Intercept)  -0.0043022  0.0006167  -6.977 2.31e-10 ***
## abs_ess      -0.0006169  0.0006412  -0.962    0.338
## gen.size.rel -0.2302102  0.0203571 -11.309  < 2e-16 ***
## ---
## Signif. codes:  0 '***' 0.001 '**' 0.01 '*' 0.05 '.' 0.1 ' ' 1
##
## Residual standard error: 0.004962 on 111 degrees of freedom
## Multiple R-squared:  0.5458, Adjusted R-squared:  0.5376
## F-statistic: 66.69 on 2 and 111 DF,  p-value: < 2.2e-16
```

```
##
## Call:
## lm(formula = rel.w ~ sum_ess + gen.size.rel, data = sc1[sc1$ploidy ==
##     "dip", ])
##
## Residuals:
##        Min         1Q     Median         3Q        Max
## -0.0189320 -0.0026074  0.0003762  0.0029247  0.0095905
##
## Coefficients:
##                Estimate Std. Error t value Pr(>|t|)
## (Intercept)  -4.240e-03  5.786e-04  -7.328    4e-11 ***
## sum_ess       1.009e-04  7.517e-05   1.342    0.182
## gen.size.rel -2.299e-01  2.023e-02 -11.364   <2e-16 ***
## ---
## Signif. codes:  0 '***' 0.001 '**' 0.01 '*' 0.05 '.' 0.1 ' ' 1
##
## Residual standard error: 0.004943 on 111 degrees of freedom
## Multiple R-squared:  0.5493, Adjusted R-squared:  0.5412
## F-statistic: 67.65 on 2 and 111 DF,  p-value: < 2.2e-16
```

```
##
## Call:
## lm(formula = rel.w ~ del + nondel + gen.size.rel, data = sc1[sc1$ploidy ==
##     "dip", ])
##
## Residuals:
##        Min         1Q     Median         3Q        Max
## -0.0189043 -0.0026875  0.0004644  0.0031718  0.0098675
##
## Coefficients:
##                Estimate Std. Error t value Pr(>|t|)
## (Intercept)  -0.0048999  0.0012805  -3.827 0.000216 ***
## del          -0.0001264  0.0002669  -0.473 0.636879
## nondel        0.0002108  0.0003187   0.661 0.509745
## gen.size.rel -0.2333729  0.0207816 -11.230  < 2e-16 ***
## ---
## Signif. codes:  0 '***' 0.001 '**' 0.01 '*' 0.05 '.' 0.1 ' ' 1
##
## Residual standard error: 0.004992 on 110 degrees of freedom
## Multiple R-squared:  0.5445, Adjusted R-squared:  0.5321
## F-statistic: 43.83 on 3 and 110 DF,  p-value: < 2.2e-16
```

```
##
## Call:
## lm(formula = rel.w ~ del_ess + nondel_ess + gen.size.rel, data = sc1[sc1$ploidy ==
##     "dip", ])
##
## Residuals:
##       Min        1Q    Median        3Q       Max
## -0.018899 -0.002275  0.000486  0.003152  0.009425
##
## Coefficients:
##                Estimate Std. Error t value Pr(>|t|)
## (Intercept)  -0.0042725  0.0007197  -5.937 3.45e-08 ***
## del_ess      -0.0008992  0.0006609  -1.361    0.176
## nondel_ess    0.0001992  0.0006769   0.294    0.769
## gen.size.rel -0.2283156  0.0204754 -11.151  < 2e-16 ***
## ---
## Signif. codes:  0 '***' 0.001 '**' 0.01 '*' 0.05 '.' 0.1 ' ' 1
##
## Residual standard error: 0.004962 on 110 degrees of freedom
## Multiple R-squared:  0.5499, Adjusted R-squared:  0.5376
## F-statistic: 44.79 on 3 and 110 DF,  p-value: < 2.2e-16
```

AIC of diploid models

```
##
## Model selection based on AIC:
##
##                K     AIC Delta_AIC AICWt Cum.Wt     LL
## n_del_ess      4 -882.20      0.00  0.19   0.19 445.10
## sum_ess        4 -882.15      0.05  0.18   0.37 445.07
## n_abs_ess      4 -881.26      0.94  0.12   0.48 444.63
```

```
## n_tot_ess      4 -880.93      1.27  0.10   0.58 444.46
## sum            4 -880.71      1.49  0.09   0.67 444.36
## n_del          4 -880.48      1.72  0.08   0.75 444.24
## n_abs          4 -880.34      1.86  0.07   0.82 444.17
## n_tot          4 -880.32      1.88  0.07   0.89 444.16
## n_nondel_ess   5 -880.29      1.91  0.07   0.96 445.14
## n_nondel       5 -878.93      3.27  0.04   1.00 444.46
## 0_n_tot        3 -868.62     13.58  0.00   1.00 437.31
## 0_sum          3 -863.77     18.43  0.00   1.00 434.89
## 0_n_del        3 -863.20     19.00  0.00   1.00 434.60
## 0_n_abs        3 -861.89     20.31  0.00   1.00 433.94
## 0_n_nondel     3 -859.72     22.48  0.00   1.00 432.86
## 0_n_tot_ess    3 -851.75     30.45  0.00   1.00 428.88
## 0_n_del_ess    3 -844.26     37.94  0.00   1.00 425.13
## 0_n_abs_ess    3 -841.81     40.39  0.00   1.00 423.90
## 0_sum_ess      3 -839.16     43.04  0.00   1.00 422.58
## 0_n_nondel_ess 3 -833.64     48.56  0.00   1.00 419.82
```

Models in Sc2

Models with intercept fixed at 0

```
##
## Call:
## lm(formula = rel.w ~ 0 + tot, data = sc2)
##
## Residuals:
##       Min        1Q    Median        3Q       Max
## -0.043006 -0.012262 -0.003042  0.005437  0.028751
##
## Coefficients:
##       Estimate Std. Error t value Pr(>|t|)
## tot -0.0010209  0.0002012  -5.074 1.04e-06 ***
## ---
## Signif. codes:  0 '***' 0.001 '**' 0.01 '*' 0.05 '.' 0.1 ' ' 1
##
## Residual standard error: 0.01436 on 165 degrees of freedom
## Multiple R-squared:  0.135,  Adjusted R-squared:  0.1297
## F-statistic: 25.74 on 1 and 165 DF,  p-value: 1.042e-06
```

```
##
## Call:
## lm(formula = rel.w ~ 0 + del, data = sc2)
##
## Residuals:
##       Min        1Q    Median        3Q       Max
## -0.045904 -0.012563 -0.004045  0.005211  0.028381
##
## Coefficients:
##       Estimate Std. Error t value Pr(>|t|)
## del -0.0017565  0.0003616  -4.858 2.74e-06 ***
## ---
```

```
## Signif. codes:  0 '***' 0.001 '**' 0.01 '*' 0.05 '.' 0.1 ' ' 1
##
## Residual standard error: 0.01444 on 165 degrees of freedom
## Multiple R-squared:  0.1251, Adjusted R-squared:  0.1198
## F-statistic:  23.6 on 1 and 165 DF,  p-value: 2.743e-06
```

```
##
## Call:
## lm(formula = rel.w ~ 0 + abs, data = sc2)
##
## Residuals:
##       Min        1Q    Median        3Q       Max
## -0.045980 -0.012601 -0.003881  0.005179  0.028356
##
## Coefficients:
##       Estimate Std. Error t value Pr(>|t|)
## abs -0.0017313  0.0003576  -4.841 2.95e-06 ***
## ---
## Signif. codes:  0 '***' 0.001 '**' 0.01 '*' 0.05 '.' 0.1 ' ' 1
##
## Residual standard error: 0.01444 on 165 degrees of freedom
## Multiple R-squared:  0.1244, Adjusted R-squared:  0.1191
## F-statistic: 23.44 on 1 and 165 DF,  p-value: 2.95e-06
```

```
##
## Call:
## lm(formula = rel.w ~ 0 + score_sum, data = sc2)
##
## Residuals:
##       Min        1Q    Median        3Q       Max
## -0.045551 -0.013526 -0.004190  0.004744  0.028234
##
## Coefficients:
##            Estimate Std. Error t value Pr(>|t|)
## score_sum 2.124e-04  4.591e-05   4.625 7.52e-06 ***
## ---
## Signif. codes:  0 '***' 0.001 '**' 0.01 '*' 0.05 '.' 0.1 ' ' 1
##
## Residual standard error: 0.01452 on 165 degrees of freedom
## Multiple R-squared:  0.1148, Adjusted R-squared:  0.1094
## F-statistic: 21.39 on 1 and 165 DF,  p-value: 7.517e-06
```

```
##
## Call:
## lm(formula = rel.w ~ 0 + tot_ess, data = sc2)
##
## Residuals:
##       Min        1Q    Median        3Q       Max
## -0.048669 -0.014542 -0.004139  0.003066  0.027636
##
## Coefficients:
##           Estimate Std. Error t value Pr(>|t|)
## tot_ess -0.0025053  0.0008102  -3.092  0.00233 **
```

```
## ---
## Signif. codes:  0 '***' 0.001 '**' 0.01 '*' 0.05 '.' 0.1 ' ' 1
##
## Residual standard error: 0.01501 on 165 degrees of freedom
## Multiple R-squared:  0.05477,    Adjusted R-squared:  0.04904
## F-statistic: 9.561 on 1 and 165 DF,  p-value: 0.002335
```

```
##
## Call:
## lm(formula = rel.w ~ 0 + del_ess, data = sc2)
##
## Residuals:
##       Min        1Q    Median        3Q       Max
## -0.047923 -0.014828 -0.005384  0.001313  0.026625
##
## Coefficients:
##          Estimate Std. Error t value Pr(>|t|)
## del_ess -0.003251   0.001270  -2.559   0.0114 *
## ---
## Signif. codes:  0 '***' 0.001 '**' 0.01 '*' 0.05 '.' 0.1 ' ' 1
##
## Residual standard error: 0.01514 on 165 degrees of freedom
## Multiple R-squared:  0.03818,    Adjusted R-squared:  0.03235
## F-statistic:  6.55 on 1 and 165 DF,  p-value: 0.01139
```

```
##
## Call:
## lm(formula = rel.w ~ 0 + sum_ess, data = sc2)
##
## Residuals:
##       Min        1Q    Median        3Q       Max
## -0.045031 -0.014948 -0.005380  0.002227  0.026625
##
## Coefficients:
##          Estimate Std. Error t value Pr(>|t|)
## sum_ess 0.0004398  0.0001659    2.65  0.00883 **
## ---
## Signif. codes:  0 '***' 0.001 '**' 0.01 '*' 0.05 '.' 0.1 ' ' 1
##
## Residual standard error: 0.01512 on 165 degrees of freedom
## Multiple R-squared:  0.04083,    Adjusted R-squared:  0.03502
## F-statistic: 7.024 on 1 and 165 DF,  p-value: 0.008826
```

```
##
## Call:
## lm(formula = rel.w ~ 0 + nondel, data = sc2)
##
## Residuals:
##       Min        1Q    Median        3Q       Max
## -0.041490 -0.012637 -0.002534  0.004822  0.030372
##
## Coefficients:
##          Estimate Std. Error t value Pr(>|t|)
```

```
## nondel -0.0019368  0.0004121  -4.699 5.47e-06 ***
## ---
## Signif. codes:  0 '***' 0.001 '**' 0.01 '*' 0.05 '.' 0.1 ' ' 1
##
## Residual standard error: 0.01449 on 165 degrees of freedom
## Multiple R-squared:  0.118,  Adjusted R-squared:  0.1127
## F-statistic: 22.09 on 1 and 165 DF,  p-value: 5.471e-06
```

```
##
## Call:
## lm(formula = rel.w ~ 0 + nondel_ess, data = sc2)
##
## Residuals:
##       Min        1Q    Median        3Q       Max
## -0.051174 -0.014948 -0.005083  0.002867  0.026642
##
## Coefficients:
##             Estimate Std. Error t value Pr(>|t|)
## nondel_ess -0.004017   0.001519  -2.644  0.00899 **
## ---
## Signif. codes:  0 '***' 0.001 '**' 0.01 '*' 0.05 '.' 0.1 ' ' 1
##
## Residual standard error: 0.01512 on 165 degrees of freedom
## Multiple R-squared:  0.04064,    Adjusted R-squared:  0.03483
## F-statistic:  6.99 on 1 and 165 DF,  p-value: 0.008989
```

Models without intercept fixed at 0

```
##
## Call:
## lm(formula = rel.w ~ tot, data = sc2)
##
## Residuals:
##       Min        1Q    Median        3Q       Max
## -0.043394 -0.008317  0.000321  0.008194  0.034155
##
## Coefficients:
##               Estimate Std. Error t value Pr(>|t|)
## (Intercept) -7.495e-03  1.521e-03  -4.927 2.02e-06 ***
## tot         -3.569e-05  2.747e-04  -0.130    0.897
## ---
## Signif. codes:  0 '***' 0.001 '**' 0.01 '*' 0.05 '.' 0.1 ' ' 1
##
## Residual standard error: 0.01344 on 164 degrees of freedom
## Multiple R-squared:  0.0001029,  Adjusted R-squared:  -0.005994
## F-statistic: 0.01688 on 1 and 164 DF,  p-value: 0.8968
```

```
##
## Call:
## lm(formula = rel.w ~ del, data = sc2)
##
## Residuals:
##       Min        1Q    Median        3Q       Max
```

```
## -0.043354 -0.008030  0.000246  0.008044  0.034054
##
## Coefficients:
##               Estimate Std. Error t value Pr(>|t|)
## (Intercept) -0.0072337  0.0014022  -5.159 7.09e-07 ***
## del         -0.0001954  0.0004525  -0.432    0.666
## ---
## Signif. codes:  0 '***' 0.001 '**' 0.01 '*' 0.05 '.' 0.1 ' ' 1
##
## Residual standard error: 0.01343 on 164 degrees of freedom
## Multiple R-squared:  0.001136,   Adjusted R-squared:  -0.004955
## F-statistic: 0.1864 on 1 and 164 DF,  p-value: 0.6665
```

```
##
## Call:
## lm(formula = rel.w ~ abs, data = sc2)
##
## Residuals:
##       Min        1Q    Median        3Q       Max
## -0.043393 -0.008013  0.000259  0.008059  0.034085
##
## Coefficients:
##               Estimate Std. Error t value Pr(>|t|)
## (Intercept) -0.0072994  0.0014124  -5.168 6.81e-07 ***
## abs         -0.0001604  0.0004506  -0.356    0.722
## ---
## Signif. codes:  0 '***' 0.001 '**' 0.01 '*' 0.05 '.' 0.1 ' ' 1
##
## Residual standard error: 0.01343 on 164 degrees of freedom
## Multiple R-squared:  0.0007722,  Adjusted R-squared:  -0.005321
## F-statistic: 0.1267 on 1 and 164 DF,  p-value: 0.7223
```

```
##
## Call:
## lm(formula = rel.w ~ score_sum, data = sc2)
##
## Residuals:
##       Min        1Q    Median        3Q       Max
## -0.043264 -0.008125  0.000195  0.008293  0.034064
##
## Coefficients:
##               Estimate Std. Error t value Pr(>|t|)
## (Intercept) -7.251e-03  1.349e-03  -5.375  2.6e-07 ***
## score_sum    2.488e-05  5.496e-05   0.453    0.651
## ---
## Signif. codes:  0 '***' 0.001 '**' 0.01 '*' 0.05 '.' 0.1 ' ' 1
##
## Residual standard error: 0.01343 on 164 degrees of freedom
## Multiple R-squared:  0.001248,   Adjusted R-squared:  -0.004841
## F-statistic: 0.205 on 1 and 164 DF,  p-value: 0.6513
```

```
##
## Call:
```

```
## lm(formula = rel.w ~ tot_ess, data = sc2)
##
## Residuals:
##       Min        1Q    Median        3Q       Max
## -0.043660 -0.008438  0.000792  0.007523  0.034729
##
## Coefficients:
##               Estimate Std. Error t value Pr(>|t|)
## (Intercept) -0.0081041  0.0012462  -6.503 9.09e-10 ***
## tot_ess      0.0005899  0.0008670   0.680    0.497
## ---
## Signif. codes:  0 '***' 0.001 '**' 0.01 '*' 0.05 '.' 0.1 ' ' 1
##
## Residual standard error: 0.01342 on 164 degrees of freedom
## Multiple R-squared:  0.002815,   Adjusted R-squared:  -0.003266
## F-statistic: 0.4629 on 1 and 164 DF,  p-value: 0.4972
```

```
##
## Call:
## lm(formula = rel.w ~ del_ess, data = sc2)
##
## Residuals:
##       Min        1Q    Median        3Q       Max
## -0.044137 -0.008395  0.000834  0.007605  0.034771
##
## Coefficients:
##              Estimate Std. Error t value Pr(>|t|)
## (Intercept) -0.008146   0.001198  -6.801 1.85e-10 ***
## del_ess      0.001109   0.001295   0.856    0.393
## ---
## Signif. codes:  0 '***' 0.001 '**' 0.01 '*' 0.05 '.' 0.1 ' ' 1
##
## Residual standard error: 0.01341 on 164 degrees of freedom
## Multiple R-squared:  0.004452,   Adjusted R-squared:  -0.001619
## F-statistic: 0.7333 on 1 and 164 DF,  p-value: 0.3931
```

```
##
## Call:
## lm(formula = rel.w ~ sum_ess, data = sc2)
##
## Residuals:
##       Min        1Q    Median        3Q       Max
## -0.044669 -0.008440  0.000693  0.007464  0.034631
##
## Coefficients:
##               Estimate Std. Error t value Pr(>|t|)
## (Intercept) -0.0080057  0.0011900  -6.728 2.74e-10 ***
## sum_ess     -0.0001075  0.0001683  -0.638    0.524
## ---
## Signif. codes:  0 '***' 0.001 '**' 0.01 '*' 0.05 '.' 0.1 ' ' 1
##
## Residual standard error: 0.01342 on 164 degrees of freedom
## Multiple R-squared:  0.002479,   Adjusted R-squared:  -0.003603
## F-statistic: 0.4076 on 1 and 164 DF,  p-value: 0.5241
```

```
##
## Call:
## lm(formula = rel.w ~ nondel, data = sc2)
##
## Residuals:
##       Min        1Q    Median        3Q       Max
## -0.043975 -0.008425  0.000609  0.007877  0.034546
##
## Coefficients:
##               Estimate Std. Error t value Pr(>|t|)
## (Intercept) -0.0079213  0.0014965  -5.293 3.81e-07 ***
## nondel       0.0001444  0.0005482   0.263    0.793
## ---
## Signif. codes:  0 '***' 0.001 '**' 0.01 '*' 0.05 '.' 0.1 ' ' 1
##
## Residual standard error: 0.01344 on 164 degrees of freedom
## Multiple R-squared:  0.0004229,  Adjusted R-squared:  -0.005672
## F-statistic: 0.06939 on 1 and 164 DF,  p-value: 0.7926
```

```
##
## Call:
## lm(formula = rel.w ~ nondel_ess, data = sc2)
##
## Residuals:
##       Min        1Q    Median        3Q       Max
## -0.043443 -0.008363  0.000418  0.007951  0.034356
##
## Coefficients:
##               Estimate Std. Error t value Pr(>|t|)
## (Intercept) -0.0077306  0.0011546  -6.695 3.26e-10 ***
## nondel_ess   0.0002779  0.0014952   0.186    0.853
## ---
## Signif. codes:  0 '***' 0.001 '**' 0.01 '*' 0.05 '.' 0.1 ' ' 1
##
## Residual standard error: 0.01344 on 164 degrees of freedom
## Multiple R-squared:  0.0002106,  Adjusted R-squared:  -0.005886
## F-statistic: 0.03455 on 1 and 164 DF,  p-value: 0.8528
```

Comparison of models using AIC

```
##
## Model selection based on AIC:
##
##             K     AIC Delta_AIC AICWt Cum.Wt     LL
## n_del_ess   3 -956.44      0.00  0.16   0.16 481.22
## n_tot_ess   3 -956.17      0.27  0.14   0.29 481.09
## sum_ess     3 -956.12      0.33  0.13   0.43 481.06
## sum         3 -955.91      0.53  0.12   0.55 480.96
## n_del       3 -955.89      0.55  0.12   0.66 480.95
## n_abs       3 -955.83      0.61  0.12   0.78 480.92
## n_nondel    3 -955.77      0.67  0.11   0.89 480.89
## n_tot       3 -955.72      0.72  0.11   1.00 480.86
## 0_n_tot     2 -934.80     21.64  0.00   1.00 469.40
```

```
## 0_n_del     2 -932.93     23.52  0.00   1.00 468.46
## 0_n_abs     2 -932.79     23.66  0.00   1.00 468.39
## 0_n_nondel  2 -931.59     24.85  0.00   1.00 467.80
## 0_sum       2 -930.98     25.47  0.00   1.00 467.49
## 0_n_tot_ess 2 -920.09     36.35  0.00   1.00 462.05
## 0_sum_ess   2 -917.66     38.78  0.00   1.00 460.83
## 0_n_del_ess 2 -917.20     39.24  0.00   1.00 460.60
```

Models in Cr

In the Cr data, we tested models using both the change in growth rate and the absolute change in growth rate. Below we note the latter models as |w|~. For each model we fit, we make a separate null model, where the fixed effect is only included as an interaction term to the random effect experienced in each ancestral background.

Models with intercept fixed at 0

In the following, we evaluate the significance of the explanatory variable by a likelihood ratio test of the model against a null, where the

```
## Linear mixed model fit by maximum likelihood . t-tests use Satterthwaite's
##   method [lmerModLmerTest]
## Formula: rel.w ~ 0 + n_tot + (0 + n_tot | anc)
##    Data: cr
##
##      AIC      BIC   logLik deviance df.resid
##   -449.4   -442.4    227.7   -455.4       72
##
## Scaled residuals:
##     Min      1Q  Median      3Q     Max
## -3.2700 -0.4367  0.0213  0.3306  1.6511
##
## Random effects:
##  Groups   Name  Variance  Std.Dev.
##  anc      n_tot 1.518e-07 0.0003896
##  Residual       1.191e-04 0.0109138
## Number of obs: 75, groups:  anc, 6
##
## Fixed effects:
##         Estimate Std. Error         df t value Pr(>|t|)
## n_tot -0.0004335  0.0001854  5.1261948  -2.339   0.0652 .
## ---
## Signif. codes:  0 '***' 0.001 '**' 0.01 '*' 0.05 '.' 0.1 ' ' 1
```

```
## Data: cr
## Models:
## crx1: rel.w ~ 0 + (0 + n_tot | anc)
## cr01: rel.w ~ 0 + n_tot + (0 + n_tot | anc)
##      npar     AIC     BIC logLik deviance  Chisq Df Pr(>Chisq)
## crx1    2 -447.50 -442.86 225.75  -451.50
## cr01    3 -449.39 -442.44 227.70  -455.39 3.8937  1    0.04847 *
```

```
## ---
## Signif. codes:  0 '***' 0.001 '**' 0.01 '*' 0.05 '.' 0.1 ' ' 1

## Linear mixed model fit by maximum likelihood . t-tests use Satterthwaite's
##   method [lmerModLmerTest]
## Formula: rel.w ~ 0 + n_del + (0 + n_del | anc)
##    Data: cr
##
##      AIC      BIC   logLik deviance df.resid
##   -455.8   -448.9    230.9   -461.8       72
##
## Scaled residuals:
##     Min      1Q  Median      3Q     Max
## -3.4709 -0.6470 -0.0555  0.3385  1.7231
##
## Random effects:
##  Groups   Name  Variance  Std.Dev.
##  anc      n_del 2.177e-06 0.001475
##  Residual       1.057e-04 0.010282
## Number of obs: 75, groups:  anc, 6
##
## Fixed effects:
##        Estimate Std. Error        df t value Pr(>|t|)
## n_del -0.001507   0.000671  5.538866  -2.246   0.0694 .
## ---
## Signif. codes:  0 '***' 0.001 '**' 0.01 '*' 0.05 '.' 0.1 ' ' 1

## Data: cr
## Models:
## crx2: rel.w ~ 0 + (0 + n_del | anc)
## cr02: rel.w ~ 0 + n_del + (0 + n_del | anc)
##      npar     AIC     BIC logLik deviance Chisq Df Pr(>Chisq)
## crx2    2 -454.01 -449.37 229.00  -458.01
## cr02    3 -455.83 -448.87 230.91  -461.83 3.817  1    0.05074 .
## ---
## Signif. codes:  0 '***' 0.001 '**' 0.01 '*' 0.05 '.' 0.1 ' ' 1

## Linear mixed model fit by maximum likelihood . t-tests use Satterthwaite's
##   method [lmerModLmerTest]
## Formula: rel.w ~ 0 + n_abs + (0 + n_abs | anc)
##    Data: cr
##
##      AIC      BIC   logLik deviance df.resid
##   -457.2   -450.2    231.6   -463.2       72
##
## Scaled residuals:
##     Min      1Q  Median      3Q     Max
## -3.5087 -0.5720 -0.0611  0.3394  1.7432
##
## Random effects:
##  Groups   Name  Variance  Std.Dev.
##  anc      n_abs 2.096e-06 0.001448
##  Residual       1.035e-04 0.010171
```

```
## Number of obs: 75, groups:  anc, 6
##
## Fixed effects:
##         Estimate Std. Error        df t value Pr(>|t|)
## n_abs -0.0014919  0.0006537  5.5590445  -2.282   0.0661 .
## ---
## Signif. codes:  0 '***' 0.001 '**' 0.01 '*' 0.05 '.' 0.1 ' ' 1
```

```
## Data: cr
## Models:
## crx3: rel.w ~ 0 + (0 + n_abs | anc)
## cr03: rel.w ~ 0 + n_abs + (0 + n_abs | anc)
##      npar     AIC     BIC logLik deviance  Chisq Df Pr(>Chisq)
## crx3    2 -455.27 -450.63 229.63  -459.27
## cr03    3 -457.16 -450.21 231.58  -463.16 3.8917  1    0.04853 *
## ---
## Signif. codes:  0 '***' 0.001 '**' 0.01 '*' 0.05 '.' 0.1 ' ' 1
```

```
## Linear mixed model fit by maximum likelihood . t-tests use Satterthwaite's
##   method [lmerModLmerTest]
## Formula: rel.w ~ 0 + logsum + (0 + logsum | anc)
##    Data: cr
##
##      AIC      BIC   logLik deviance df.resid
##   -464.0   -457.1    235.0   -470.0       72
##
## Scaled residuals:
##     Min      1Q  Median      3Q     Max
## -3.3865 -0.3451  0.0893  0.4197  1.5946
##
## Random effects:
##  Groups   Name   Variance  Std.Dev.
##  anc      logsum 1.296e-06 0.001139
##  Residual        9.412e-05 0.009701
## Number of obs: 75, groups:  anc, 6
##
## Fixed effects:
##          Estimate Std. Error        df t value Pr(>|t|)
## logsum -0.0011155  0.0004976  5.9403649  -2.242   0.0666 .
## ---
## Signif. codes:  0 '***' 0.001 '**' 0.01 '*' 0.05 '.' 0.1 ' ' 1
```

```
## Data: cr
## Models:
## crx4: rel.w ~ 0 + (0 + logsum | anc)
## cr04: rel.w ~ 0 + logsum + (0 + logsum | anc)
##      npar     AIC     BIC logLik deviance Chisq Df Pr(>Chisq)
## crx4    2 -462.34 -457.70 233.17  -466.34
## cr04    3 -464.00 -457.05 235.00  -470.00 3.667  1     0.0555 .
## ---
## Signif. codes:  0 '***' 0.001 '**' 0.01 '*' 0.05 '.' 0.1 ' ' 1
```

```
## Linear mixed model fit by maximum likelihood . t-tests use Satterthwaite's
```

```
##   method [lmerModLmerTest]
## Formula: rel.w ~ 0 + nondel + (0 + nondel | anc)
##    Data: cr
##
##      AIC      BIC   logLik deviance df.resid
##   -455.8   -448.9    230.9   -461.8       72
##
## Scaled residuals:
##     Min      1Q  Median      3Q     Max
## -3.4709 -0.6470 -0.0555  0.3385  1.7231
##
## Random effects:
##  Groups   Name   Variance  Std.Dev.
##  anc      nondel 2.177e-06 0.001475
##  Residual        1.057e-04 0.010282
## Number of obs: 75, groups:  anc, 6
##
## Fixed effects:
##         Estimate Std. Error        df t value Pr(>|t|)
## nondel -0.001507   0.000671  5.538866  -2.246   0.0694 .
## ---
## Signif. codes:  0 '***' 0.001 '**' 0.01 '*' 0.05 '.' 0.1 ' ' 1
```

```
## Data: cr
## Models:
## crx5: rel.w ~ 0 + (0 + nondel | anc)
## cr05: rel.w ~ 0 + nondel + (0 + nondel | anc)
##      npar     AIC     BIC logLik deviance Chisq Df Pr(>Chisq)
## crx5    2 -454.01 -449.37 229.00  -458.01
## cr05    3 -455.83 -448.87 230.91  -461.83 3.817  1    0.05074 .
## ---
## Signif. codes:  0 '***' 0.001 '**' 0.01 '*' 0.05 '.' 0.1 ' ' 1
```

```
## Linear mixed model fit by maximum likelihood . t-tests use Satterthwaite's
##   method [lmerModLmerTest]
## Formula: abs(rel.w) ~ 0 + n_tot + (0 + n_tot | anc)
##    Data: cr
##
##      AIC      BIC   logLik deviance df.resid
##   -456.7   -449.8    231.4   -462.7       72
##
## Scaled residuals:
##     Min      1Q  Median      3Q     Max
## -1.4181 -0.1910  0.0745  0.7243  3.3601
##
## Random effects:
##  Groups   Name  Variance  Std.Dev.
##  anc      n_tot 6.338e-08 0.0002517
##  Residual       1.128e-04 0.0106214
## Number of obs: 75, groups:  anc, 6
##
## Fixed effects:
##        Estimate Std. Error        df t value Pr(>|t|)
## n_tot 0.0005589  0.0001347 7.1310413   4.149  0.00413 **
```

```
## ---
## Signif. codes:  0 '***' 0.001 '**' 0.01 '*' 0.05 '.' 0.1 ' ' 1

## Data: cr
## Models:
## crx6: abs(rel.w) ~ 0 + (0 + n_tot | anc)
## cr06: abs(rel.w) ~ 0 + n_tot + (0 + n_tot | anc)
##      npar     AIC     BIC logLik deviance  Chisq Df Pr(>Chisq)
## crx6    2 -449.44 -444.80 226.72  -453.44
## cr06    3 -456.72 -449.76 231.36  -462.72 9.2804  1   0.002316 **
## ---
## Signif. codes:  0 '***' 0.001 '**' 0.01 '*' 0.05 '.' 0.1 ' ' 1

## Linear mixed model fit by maximum likelihood . t-tests use Satterthwaite's
##   method [lmerModLmerTest]
## Formula: abs(rel.w) ~ 0 + n_del + (0 + n_del | anc)
##    Data: cr
##
##      AIC      BIC   logLik deviance df.resid
##   -461.9   -454.9    233.9   -467.9       72
##
## Scaled residuals:
##     Min      1Q  Median      3Q     Max
## -1.2335 -0.2216  0.1385  0.7807  3.5293
##
## Random effects:
##  Groups   Name  Variance  Std.Dev.
##  anc      n_del 9.513e-07 0.0009753
##  Residual       1.023e-04 0.0101123
## Number of obs: 75, groups:  anc, 6
##
## Fixed effects:
##       Estimate Std. Error       df t value Pr(>|t|)
## n_del 0.001871   0.000483 6.137991   3.873  0.00788 **
## ---
## Signif. codes:  0 '***' 0.001 '**' 0.01 '*' 0.05 '.' 0.1 ' ' 1

## Data: cr
## Models:
## crx7: abs(rel.w) ~ 0 + (0 + n_del | anc)
## cr07: abs(rel.w) ~ 0 + n_del + (0 + n_del | anc)
##      npar     AIC     BIC logLik deviance  Chisq Df Pr(>Chisq)
## crx7    2 -455.53 -450.90 229.77  -459.53
## cr07    3 -461.88 -454.93 233.94  -467.88 8.3459  1   0.003866 **
## ---
## Signif. codes:  0 '***' 0.001 '**' 0.01 '*' 0.05 '.' 0.1 ' ' 1

## Linear mixed model fit by maximum likelihood . t-tests use Satterthwaite's
##   method [lmerModLmerTest]
## Formula: abs(rel.w) ~ 0 + n_abs + (0 + n_abs | anc)
##    Data: cr
##
##      AIC      BIC   logLik deviance df.resid
```

```
##   -463.2   -456.2    234.6   -469.2       72
##
## Scaled residuals:
##     Min      1Q  Median      3Q     Max
## -1.1758 -0.2314  0.1503  0.7569  3.5663
##
## Random effects:
##  Groups   Name  Variance  Std.Dev.
##  anc      n_abs 9.208e-07 0.0009596
##  Residual       1.001e-04 0.0100071
## Number of obs: 75, groups:  anc, 6
##
## Fixed effects:
##        Estimate Std. Error        df t value Pr(>|t|)
## n_abs 0.0018425  0.0004701 6.1388753    3.92  0.00746 **
## ---
## Signif. codes:  0 '***' 0.001 '**' 0.01 '*' 0.05 '.' 0.1 ' ' 1
```

```
## Data: cr
## Models:
## crx8: abs(rel.w) ~ 0 + (0 + n_abs | anc)
## cr08: abs(rel.w) ~ 0 + n_abs + (0 + n_abs | anc)
##      npar    AIC     BIC logLik deviance  Chisq Df Pr(>Chisq)
## crx8    2 -456.8 -452.17  230.4   -460.8
## cr08    3 -463.2 -456.25  234.6   -469.2 8.4007  1   0.003751 **
## ---
## Signif. codes:  0 '***' 0.001 '**' 0.01 '*' 0.05 '.' 0.1 ' ' 1
```

```
## Linear mixed model fit by maximum likelihood . t-tests use Satterthwaite's
##   method [lmerModLmerTest]
## Formula: abs(rel.w) ~ 0 + logsum + (0 + logsum | anc)
##    Data: cr
##
##      AIC      BIC   logLik deviance df.resid
##   -473.1   -466.2    239.6   -479.1       72
##
## Scaled residuals:
##     Min      1Q  Median      3Q     Max
## -0.9556 -0.3595 -0.0995  0.5801  3.5090
##
## Random effects:
##  Groups   Name   Variance  Std.Dev.
##  anc      logsum 4.497e-07 0.0006706
##  Residual        8.890e-05 0.0094285
## Number of obs: 75, groups:  anc, 6
##
## Fixed effects:
##         Estimate Std. Error        df t value Pr(>|t|)
## logsum 0.0014745  0.0003232 5.6958948   4.562  0.00438 **
## ---
## Signif. codes:  0 '***' 0.001 '**' 0.01 '*' 0.05 '.' 0.1 ' ' 1
```

```
## Data: cr
```

```
## Models:
## crx9: abs(rel.w) ~ 0 + (0 + logsum | anc)
## cr09: abs(rel.w) ~ 0 + logsum + (0 + logsum | anc)
##      npar     AIC     BIC logLik deviance  Chisq Df Pr(>Chisq)
## crx9    2 -466.14 -461.51 235.07  -470.14
## cr09    3 -473.11 -466.16 239.55  -479.11 8.9679  1   0.002748 **
## ---
## Signif. codes:  0 '***' 0.001 '**' 0.01 '*' 0.05 '.' 0.1 ' ' 1
```

```
## Linear mixed model fit by maximum likelihood . t-tests use Satterthwaite's
##   method [lmerModLmerTest]
## Formula: abs(rel.w) ~ 0 + nondel + (0 + nondel | anc)
##    Data: cr
##
##      AIC      BIC   logLik deviance df.resid
##   -461.9   -454.9    233.9   -467.9       72
##
## Scaled residuals:
##     Min      1Q  Median      3Q     Max
## -1.2335 -0.2216  0.1385  0.7807  3.5293
##
## Random effects:
##  Groups   Name   Variance  Std.Dev.
##  anc      nondel 9.513e-07 0.0009753
##  Residual        1.023e-04 0.0101123
## Number of obs: 75, groups:  anc, 6
##
## Fixed effects:
##        Estimate Std. Error       df t value Pr(>|t|)
## nondel 0.001871   0.000483 6.137991   3.873  0.00788 **
## ---
## Signif. codes:  0 '***' 0.001 '**' 0.01 '*' 0.05 '.' 0.1 ' ' 1
```

```
## Data: cr
## Models:
## crx010: abs(rel.w) ~ 0 + (0 + nondel | anc)
## cr010: abs(rel.w) ~ 0 + nondel + (0 + nondel | anc)
##        npar     AIC     BIC logLik deviance  Chisq Df Pr(>Chisq)
## crx010    2 -455.53 -450.90 229.77  -459.53
## cr010     3 -461.88 -454.93 233.94  -467.88 8.3459  1   0.003866 **
## ---
## Signif. codes:  0 '***' 0.001 '**' 0.01 '*' 0.05 '.' 0.1 ' ' 1
```

Models without intercept fixed at 0

```
## Linear mixed model fit by maximum likelihood . t-tests use Satterthwaite's
##   method [lmerModLmerTest]
## Formula: rel.w ~ n_tot + (n_tot | anc)
##    Data: cr
##
##      AIC      BIC   logLik deviance df.resid
##   -497.1   -483.2    254.5   -509.1       69
##
```

```
## Scaled residuals:
##     Min      1Q  Median      3Q     Max
## -2.3487 -0.5290  0.1888  0.6335  2.1256
##
## Random effects:
##  Groups   Name        Variance  Std.Dev.  Corr
##  anc      (Intercept) 1.221e-04 0.0110496
##           n_tot       3.884e-08 0.0001971 -1.00
##  Residual             5.269e-05 0.0072590
## Number of obs: 75, groups:  anc, 6
##
## Fixed effects:
##              Estimate Std. Error        df t value Pr(>|t|)
## (Intercept) -0.0104130  0.0048105  6.3916198  -2.165   0.0708 .
## n_tot        0.0001308  0.0001191  9.6092019   1.098   0.2990
## ---
## Signif. codes:  0 '***' 0.001 '**' 0.01 '*' 0.05 '.' 0.1 ' ' 1
##
## Correlation of Fixed Effects:
##       (Intr)
## n_tot -0.853
## convergence code: 0
## boundary (singular) fit: see ?isSingular
```

```
## Linear mixed model fit by maximum likelihood . t-tests use Satterthwaite's
##   method [lmerModLmerTest]
## Formula: rel.w ~ n_del + (n_del | anc)
##    Data: cr
##
##      AIC      BIC   logLik deviance df.resid
##   -491.5   -477.6    251.8   -503.5       69
##
## Scaled residuals:
##     Min      1Q  Median      3Q     Max
## -2.3064 -0.4683  0.1582  0.6649  1.9878
##
## Random effects:
##  Groups   Name        Variance  Std.Dev.  Corr
##  anc      (Intercept) 7.699e-05 0.0087746
##           n_del       3.575e-09 0.0000598 -1.00
##  Residual             5.673e-05 0.0075316
## Number of obs: 75, groups:  anc, 6
##
## Fixed effects:
##              Estimate Std. Error        df t value Pr(>|t|)
## (Intercept) -8.597e-03  3.944e-03  6.675e+00  -2.180   0.0676 .
## n_del        7.201e-05  2.891e-04  6.573e+01   0.249   0.8041
## ---
## Signif. codes:  0 '***' 0.001 '**' 0.01 '*' 0.05 '.' 0.1 ' ' 1
##
## Correlation of Fixed Effects:
##       (Intr)
## n_del -0.431
## convergence code: 0
```

```
## boundary (singular) fit: see ?isSingular

## Linear mixed model fit by maximum likelihood . t-tests use Satterthwaite's
##   method [lmerModLmerTest]
## Formula: rel.w ~ n_abs + (n_abs | anc)
##    Data: cr
##
##      AIC      BIC   logLik deviance df.resid
##   -491.5   -477.6    251.7   -503.5       69
##
## Scaled residuals:
##     Min      1Q  Median      3Q     Max
## -2.2934 -0.4676  0.1729  0.6630  1.9895
##
## Random effects:
##  Groups   Name        Variance  Std.Dev.  Corr
##  anc      (Intercept) 7.808e-05 8.836e-03
##           n_abs       5.725e-09 7.567e-05 -1.00
##  Residual             5.677e-05 7.535e-03
## Number of obs: 75, groups:  anc, 6
##
## Fixed effects:
##               Estimate Std. Error         df t value Pr(>|t|)
## (Intercept) -8.391e-03  3.970e-03  6.679e+00  -2.114   0.0743 .
## n_abs        2.797e-05  2.815e-04  7.163e+01   0.099   0.9211
## ---
## Signif. codes:  0 '***' 0.001 '**' 0.01 '*' 0.05 '.' 0.1 ' ' 1
##
## Correlation of Fixed Effects:
##       (Intr)
## n_abs -0.452
## convergence code: 0
## boundary (singular) fit: see ?isSingular

## Linear mixed model fit by maximum likelihood . t-tests use Satterthwaite's
##   method [lmerModLmerTest]
## Formula: rel.w ~ logsum + (logsum | anc)
##    Data: cr
##
##      AIC      BIC   logLik deviance df.resid
##   -495.8   -481.9    253.9   -507.8       69
##
## Scaled residuals:
##      Min       1Q   Median       3Q      Max
## -2.54594 -0.51585  0.09561  0.69970  2.15156
##
## Random effects:
##  Groups   Name        Variance  Std.Dev.  Corr
##  anc      (Intercept) 1.286e-04 0.0113399
##           logsum      2.322e-07 0.0004819 -1.00
##  Residual             5.329e-05 0.0072998
## Number of obs: 75, groups:  anc, 6
##
## Fixed effects:
```

```
##               Estimate Std. Error         df t value Pr(>|t|)
## (Intercept) -0.0122103  0.0052597  6.8559926  -2.321    0.054 .
## logsum       0.0006522  0.0004337 15.4447056   1.504    0.153
## ---
## Signif. codes:  0 '***' 0.001 '**' 0.01 '*' 0.05 '.' 0.1 ' ' 1
##
## Correlation of Fixed Effects:
##        (Intr)
## logsum -0.797
## convergence code: 0
## boundary (singular) fit: see ?isSingular

## Linear mixed model fit by maximum likelihood . t-tests use Satterthwaite's
##   method [lmerModLmerTest]
## Formula: rel.w ~ nondel + (nondel | anc)
##    Data: cr
##
##      AIC      BIC   logLik deviance df.resid
##   -491.5   -477.6    251.8   -503.5       69
##
## Scaled residuals:
##     Min      1Q  Median      3Q     Max
## -2.3064 -0.4683  0.1582  0.6649  1.9878
##
## Random effects:
##  Groups   Name        Variance  Std.Dev.  Corr
##  anc      (Intercept) 7.699e-05 0.0087746
##           nondel      3.575e-09 0.0000598 -1.00
##  Residual             5.673e-05 0.0075316
## Number of obs: 75, groups:  anc, 6
##
## Fixed effects:
##               Estimate Std. Error         df t value Pr(>|t|)
## (Intercept) -8.597e-03  3.944e-03  6.675e+00  -2.180   0.0676 .
## nondel       7.201e-05  2.891e-04  6.573e+01   0.249   0.8041
## ---
## Signif. codes:  0 '***' 0.001 '**' 0.01 '*' 0.05 '.' 0.1 ' ' 1
##
## Correlation of Fixed Effects:
##        (Intr)
## nondel -0.431
## convergence code: 0
## boundary (singular) fit: see ?isSingular

## Linear mixed model fit by maximum likelihood . t-tests use Satterthwaite's
##   method [lmerModLmerTest]
## Formula: abs(rel.w) ~ n_tot + (n_tot | anc)
##    Data: cr
##
##      AIC      BIC   logLik deviance df.resid
##   -520.0   -506.1    266.0   -532.0       69
##
## Scaled residuals:
##     Min      1Q  Median      3Q     Max
```

```
## -2.3835 -0.6470 -0.1563  0.4561  2.6752
##
## Random effects:
##  Groups   Name        Variance  Std.Dev.  Corr
##  anc      (Intercept) 5.659e-05 0.0075227
##           n_tot       1.946e-08 0.0001395 -1.00
##  Residual             4.034e-05 0.0063512
## Number of obs: 75, groups:  anc, 6
##
## Fixed effects:
##               Estimate Std. Error         df t value Pr(>|t|)
## (Intercept)  1.389e-02  3.393e-03  6.657e+00   4.095  0.00512 **
## n_tot       -2.028e-04  9.458e-05  1.438e+01  -2.145  0.04951 *
## ---
## Signif. codes:  0 '***' 0.001 '**' 0.01 '*' 0.05 '.' 0.1 ' ' 1
##
## Correlation of Fixed Effects:
##       (Intr)
## n_tot -0.834
## convergence code: 0
## boundary (singular) fit: see ?isSingular

## Linear mixed model fit by maximum likelihood . t-tests use Satterthwaite's
##   method [lmerModLmerTest]
## Formula: abs(rel.w) ~ n_del + (n_del | anc)
##    Data: cr
##
##      AIC      BIC   logLik deviance df.resid
##   -512.2   -498.3    262.1   -524.2       69
##
## Scaled residuals:
##     Min      1Q  Median      3Q     Max
## -2.1796 -0.6765 -0.2112  0.4241  2.7097
##
## Random effects:
##  Groups   Name        Variance  Std.Dev.  Corr
##  anc      (Intercept) 3.410e-05 5.839e-03
##           n_del       1.922e-09 4.383e-05 -1.00
##  Residual             4.493e-05 6.703e-03
## Number of obs: 75, groups:  anc, 6
##
## Fixed effects:
##               Estimate Std. Error         df t value Pr(>|t|)
## (Intercept)  0.0117961  0.0027891  6.7985172   4.229  0.00416 **
## n_del       -0.0001934  0.0002518 54.6778013  -0.768  0.44566
## ---
## Signif. codes:  0 '***' 0.001 '**' 0.01 '*' 0.05 '.' 0.1 ' ' 1
##
## Correlation of Fixed Effects:
##       (Intr)
## n_del -0.498
## convergence code: 0
## boundary (singular) fit: see ?isSingular
```

```
## Linear mixed model fit by maximum likelihood . t-tests use Satterthwaite's
##   method [lmerModLmerTest]
## Formula: abs(rel.w) ~ n_abs + (n_abs | anc)
##    Data: cr
##
##      AIC      BIC   logLik deviance df.resid
##   -512.1   -498.2    262.1   -524.1       69
##
## Scaled residuals:
##     Min      1Q  Median      3Q     Max
## -2.1834 -0.6753 -0.2263  0.3840  2.7135
##
## Random effects:
##  Groups   Name        Variance  Std.Dev. Corr
##  anc      (Intercept) 3.609e-05 0.006008
##           n_abs       5.777e-09 0.000076 -1.00
##  Residual             4.501e-05 0.006709
## Number of obs: 75, groups:  anc, 6
##
## Fixed effects:
##               Estimate Std. Error         df t value Pr(>|t|)
## (Intercept)  0.0116133  0.0028509  6.7980506   4.074  0.00503 **
## n_abs       -0.0001533  0.0002446 68.2187554  -0.627  0.53282
## ---
## Signif. codes:  0 '***' 0.001 '**' 0.01 '*' 0.05 '.' 0.1 ' ' 1
##
## Correlation of Fixed Effects:
##       (Intr)
## n_abs -0.536
## convergence code: 0
## boundary (singular) fit: see ?isSingular
```

```
## Linear mixed model fit by maximum likelihood . t-tests use Satterthwaite's
##   method [lmerModLmerTest]
## Formula: abs(rel.w) ~ logsum + (logsum | anc)
##    Data: cr
##
##      AIC      BIC   logLik deviance df.resid
##   -520.2   -506.3    266.1   -532.2       69
##
## Scaled residuals:
##     Min      1Q  Median      3Q     Max
## -2.4492 -0.6804 -0.1513  0.4275  3.0716
##
## Random effects:
##  Groups   Name        Variance  Std.Dev.  Corr
##  anc      (Intercept) 7.705e-05 0.0087780
##           logsum      2.887e-07 0.0005373 -1.00
##  Residual             4.008e-05 0.0063308
## Number of obs: 75, groups:  anc, 6
##
## Fixed effects:
##               Estimate Std. Error         df t value Pr(>|t|)
## (Intercept)  0.0155688  0.0041996  6.6967242   3.707  0.00821 **
```

```
## logsum      -0.0007828  0.0004022 10.7320238  -1.946  0.07827 .
## ---
## Signif. codes:  0 '***' 0.001 '**' 0.01 '*' 0.05 '.' 0.1 ' ' 1
##
## Correlation of Fixed Effects:
##        (Intr)
## logsum -0.877
## convergence code: 0
## boundary (singular) fit: see ?isSingular
```

```
## Linear mixed model fit by maximum likelihood . t-tests use Satterthwaite's
##   method [lmerModLmerTest]
## Formula: abs(rel.w) ~ nondel + (nondel | anc)
##    Data: cr
##
##      AIC      BIC   logLik deviance df.resid
##   -512.2   -498.3    262.1   -524.2       69
##
## Scaled residuals:
##     Min      1Q  Median      3Q     Max
## -2.1796 -0.6765 -0.2112  0.4241  2.7097
##
## Random effects:
##  Groups   Name        Variance  Std.Dev.  Corr
##  anc      (Intercept) 3.410e-05 5.839e-03
##           nondel      1.922e-09 4.383e-05 -1.00
##  Residual             4.493e-05 6.703e-03
## Number of obs: 75, groups:  anc, 6
##
## Fixed effects:
##               Estimate Std. Error         df t value Pr(>|t|)
## (Intercept)  0.0117961  0.0027891  6.7985172   4.229  0.00416 **
## nondel      -0.0001934  0.0002518 54.6778013  -0.768  0.44566
## ---
## Signif. codes:  0 '***' 0.001 '**' 0.01 '*' 0.05 '.' 0.1 ' ' 1
##
## Correlation of Fixed Effects:
##        (Intr)
## nondel -0.498
## convergence code: 0
## boundary (singular) fit: see ?isSingular
```

Comparison of models using AIC

```
## Warning in aictab.AIClmerModLmerTest(list(cr01, cr02, cr03, cr04, cr05, :
## Check model structure carefully as some models may be redundant
```

```
##
## Model selection based on AIC:
##
##          K     AIC Delta_AIC AICWt Cum.Wt     LL
## n_tot    6 -497.06      0.00  0.58   0.58 254.53
## sum      6 -495.85      1.21  0.31   0.89 253.92
```

```
## n_del    6 -491.52      5.54  0.04   0.93 251.76
## n_nondel 6 -491.52      5.54  0.04   0.96 251.76
## n_abs    6 -491.50      5.56  0.04   1.00 251.75
## 0_sum    3 -464.00     33.05  0.00   1.00 235.00
## 0_n_abs  3 -457.16     39.90  0.00   1.00 231.58
## 0_n_del  3 -455.83     41.23  0.00   1.00 230.91
## 0_nondel 3 -455.83     41.23  0.00   1.00 230.91
## 0_n_tot  3 -449.39     47.67  0.00   1.00 227.70

## Warning in aictab.AIClmerModLmerTest(list(cr06, cr07, cr08, cr09, cr010, :
## Check model structure carefully as some models may be redundant

##
## Model selection based on AIC:
##
##                K     AIC Delta_AIC AICWt Cum.Wt     LL
## |w|~sum        6 -520.17      0.00  0.51   0.51 266.08
## |w|~n_tot      6 -520.00      0.16  0.47   0.97 266.00
## |w|~n_del      6 -512.25      7.92  0.01   0.98 262.12
## |w|~n_nondel   6 -512.25      7.92  0.01   0.99 262.12
## |w|~n_abs      6 -512.12      8.05  0.01   1.00 262.06
## |w|~0_sum      3 -473.11     47.06  0.00   1.00 239.55
## |w|~0_n_abs    3 -463.20     56.97  0.00   1.00 234.60
## |w|~0_n_del    3 -461.88     58.29  0.00   1.00 233.94
## |w|~0_n_nondel 3 -461.88     58.29  0.00   1.00 233.94
## |w|~0_n_tot    3 -456.72     63.45  0.00   1.00 231.36
```
