# Supplementary material for: Fitness Effects of Mutations: An Assessment of PROVEAN Predictions Using Mutation Accumulation Data
Source: Genome Biol Evol. 2022 Jan 17;14(1):evac004. doi: 10.1093/gbe/evac004 (PMC8790079; doi:10.1093/gbe/evac004)
Supplement: evac004_Supplementary_Data [file evac004_supplementary_data.zip › TableS1.docx]

Table S1 The use of PROVEAN is widespread in ecology and evolutionary studies. The table summarizes 72 research articles that uses PROVEAN to gauge the impact of protein variants in non-human species. The default cutoff score for calling protein variants deleterious in PROVEAN is –2.5. The number of proteins in some cases had to be estimated, as we were unable to find any specific number in the article. The trait/pattern column categorizes the studies based on research topic and aim.

| **Reference** | **Species** | **Cutoff score** | **Number of proteins** | **Trait/pattern** |
| --- | --- | --- | --- | --- |
| Al Khatib, Al Thani, & Yassine, 2018 | H1N1 influenza | default | 1 | Protein evolution |
| Al-Shuhaib et al., 2018 | Ostriches | default | 6 | Find candidate loci for adaptation |
| Alavijeh et al., 2020 | Citrus red mite, *Panonychus citri* | default | 1 | Resistance to insecticide |
| Alfano et al., 2020 | Tick-borne encephalitis (TBE) | default | 1 | Assess variants compared to reference strain |
| Alvarez, Castellano, Recio, & Cabrera, 2019 | Wild barley, *Hordeum chilense* | default | 1 | Amylose synthesis |
| Bajda et al., 2017 | Red spider mite, *Tetranychus urticae* | default | 1 | Resistance to insecticide |
| Bazhenov, Chernook, Kroupin, Karlov, & Divashuk, 2020 | Wild grass, *Dasypyrum villosum* | not reported | 1 | Assess allelic diveristy |
| Bhattacharya, Dhar, Banerjee, & Ray, 2019 | Common wheat, *Triticum aestivum* | default | 1 | Assess functional impacts of differences between two consensus domains of protein |
| Buhl et al., 2019 | *Pseudomonas aeruginosa* | default | 21 | Find candidate variant for resistance |
| Cabrera, Castellano, Recio, & Alvarez, 2019 | Crested wheat grass, *Agropyron cristatum* | not reported | 3 | Grain hardness |
| Castellá, Bragulat, Cigliano, & Cabañes, 2020 | *Aspergillus carbonarius* | not reported | 1 | Assess variation within species |
| Cheng et al., 2017 | Soybean | not reported | 14 | Resistance to fungi *Fusarium graminearum* |
| Conte et al., 2017 | Spruce | default (but tests others) | 6928 | Estimate mutation load |
| Danaher et al., 2017 | HSV-1 | not reported | 64 | Find variants associated with plaque size |
| De Lomana et al., 2017 | Yeast, *Saccharomyces cerevisiae* | not reported | 2 | Assess adaptations under experimental evolution |
| del Olmo, Poza-Viejo, Piñeiro, Jarillo, & Crevillén, 2019 | *Brassica rapa* | default | 1 | Find candidate variants for flowering time |
| Diaz Caballero et al., 2018 | Proteobacteria, *Burkholderia multivorans* | not reported | 62 | Antibiotic resistance |
| Ferchaud, Laporte, Perrier, & Bernatchez, 2018 | Lake trout, *Salvelinus namaycush* | default | ~4000 | Assess mutational load |
| Fisher, Kryazhimskiy, & Lang, 2019 | Budding yeast, *Saccharomyces cerevisiae* | not reported | 9 | Describe adaptive mutations from experimental evolution |
| Gammerdinger, Conte, Sandkam, Penman, & Kocher, 2019 | Cichlids, Pseudocrenilabrinae | default | >600 | Sex-determination |
| Gorter et al., 2017 | Yeast, *Saccharomyces cerevisiae* | default | ~200 | Assess adaptations under experimental evolution |
| Hall, Harrison, & Brockhurst, 2018 | Pseudomonas spp. | not reported | 1 | Assess variation among species; candidate variants for trait variation |
| Hamabata et al., 2019 | Endangered island endemic plants | abs(2.5) | Tens of thousands | Assess mutational load |
| Harrison, Guymer, Spiers, Paterson, & Brockhurst, 2015 | *Pseudomonas fluorescens* | not reported | 2 | Find adaptative variants from experimental evolution |
| He et al., 2019 | Vampire bats, Phyllostomidae | default | 1 | Find candidate variants for immune system adaptation |
| Henson et al., 2017 | Red wolf and Maned wolf | default | 1 | Intestinal bowel disease |
| Heuermann et al., 2019 | Zea maize | not reported | 2 | Find causal variants for mutant phenotypes |
| Hodgins et al., 2015 | Asteraceae | default | 100s | Estimate mutational load between species |
| Kadian et al., 2018 | *Plasmodium vivax* | default | 1 | Protein evolution |
| Kardos, Taylor, Ellegren, Luikart, & Allendorf, 2016 | Review | na | na | Assess inbreeding depression |
| Khalid, Khalid, Gul, Amir, & Ahmad, 2018 | Common wheat, *Triticum aestivum* | default | 1 | Drought tolerance |
| Klimushina, Kroupin, Bazhenov, Karlov, & Divashuk, 2020 | Common wheat, *Triticum aestivum* | default | 7 (orthologs) | Amylose synthesis |
| Kusakabe et al., 2017 | Stickleback, *Gasterosteus aculeatus* | abs(2.5) | 530 | Filter QTL candidates for adaptative differences between ecotypes |
| Lecová, Tůmová, & Nohýnková, 2019 | *Giardia intestinalis* | default | 2 | Population structure |
| Li et al., 2014 | Antarctic penguin | default | 4922 | Find candidate genes for adaptation |
| Lind, Arvidsson, Berg, & Andersson, 2017 | *Salmonella typhimurium* | not reported | 3 | Classifying mutational effects of proteins in bacteria and relating to competitive fitness |
| Liu, Zhou, Morrell, & Gaut, 2017 | Rice, *Oryza sativa* | not reported | thousands | Assess mutational load |
| Longo et al., 2019 | *Klebsiella pneumoniae* | default | 6 | Find candidate variant for colistin resistance |
| Makino et al., 2018 | Multiple domesticated species | default | Tens of thousands | Assess mutational load |
| Martino et al., 2018 | *Lactobacillus plantarum* | default | 3 | Assess function of candidate variants for adaptation |
| Mercatanti, Lodovichi, Cervelli, & Galli, 2017 | Yeast, *Saccharomyces cerevisiae* | not reported | NA | Human cancer-associated variants |
| Morales, Pavlova, Joseph, & Sunnucks, 2015 | Yellow robin, *Eopsaltria australis* | -4.1 | 12 | Evaluate candidate variants for mitochondrial lineage differences |
| Navarro-Sigüenza, Vázquez-Miranda, Hernández-Alonso, García-Trejo, & Sánchez-González, 2017 | Woodpecker, *Melanerpes* | default | 6 | Assess variation among species |
| Ochoa, Onorato, Fitak, Roelke-Parker, & Culver, 2017 | Florida panthers and Texas puma | default | 2 | Screening for mutations deleterious for species |
| Ofori-Anyinam et al., 2020 | *Mycobacterium africanum, Mycobacterium tuberculosis* | default | 18 | Assess candidate variants between lineages for metabolic differences |
| Parker, Berny Mier y Teran, Palkovic, Jernstedt, & Gepts, 2020 | Common bean, *Phaseolus vulgaris* | default | 1 | Functional importance of candidate variant for pod indehiscence |
| Perrier et al., 2017 | Lake trout, *Salvelinus namaycush* | default | 207 | Estimate mutational load within species |
| Piombo, Bosio, Acquadro, Abbruscato, & Spadaro, 2020 | Hemibiotrophic fungus *Fusarium fujikuroi* | not reported | 36 | Find variants for trait variation |
| Priyam, Tripathy, Rai, & Ghorai, 2018 | Multiple reptilian species | default | 4 | Assess variation among species |
| Rasal et al., 2016 | Zebrafish, *Danio rerio* | default | 1 | Single gene analysis |
| Renaut & Rieseberg, 2015 | Compositae crops | default | >1000? | Estimate mutational load in domesticated species |
| Rowland et al., 2020 | Tomato, *Solanum lycopersicum* | default | 1 | Find variants for trait variation |
| Schweizer et al., 2016 | Grey wolves | not reported | 1 | Functional impact on protein |
| Seung, Echevarría-Poza, Steuernagel, & Smith, 2020 | *Arabidopsis thaliana* | not reported | 1 | Filter sequence variants for functional effects |
| Sharma et al., 2018 | HIV-1 | default | 1 | Protein evolution |
| Slugina, Shchennikova, Pishnaya, & Kochieva, 2018 | Tomato, *Solanum lycopersicum* | not reported | 15 (orthologs) | Self-compatibility |
| Stage et al., 2020 | *Lactobacillus rhamnosus* | not reported | 6 | Assess phenotypic impact of SNPs |
| Sun et al., 2019 | Small brown planthoppers, *Laodelphax striatellus* | default | 3 | Climate adaptation (cold tolerance) |
| Takahashi et al., 2017 | Experimental mouse | not reported | 1 | Cataract |
| Tancos et al., 2020 | *Rathayibacter toxicus* | default | 20 | Assess functionality of antibiotic genes |
| Vázquez-Miranda et al., 2017 | Le Conte's thrasher *Toxostoma lecontei* | default | 17 | Plumage color |
| Veilleux, Louis, & Bolnick, 2013 | Nocturnal lemurs | not reported | 1 | Assess variation among species; estimate mutational load |
| Wang et al., 2020 | Fruit bats | -1.3 | 878 (orthologs) | Convergent evolution of frugivory |
| Yakubu, Salako, De Donato, & Imumorin, 2017 | Nigerian goats | default | 1 | Detect potential sites under positive selection |
| Yoshida et al., 2016 | Stickleback, *Gasterosteus aculeatus*, *G. nipponicus* | abs(1.3),abs(2.5), abs(4.1) | 38 | Find functional differences between proteins in two different species |
| Yoshida et al., 2019 | Stickleback, *Gasterosteus aculeatus* | abs(2.5) | 1 | Hybrid male sterility |
| Yoshida et al., 2020 | Threespine stickleback, *Gasterosteus aculeatus* | default | 3512 | Assess mutational load |
| Yoshida, Makino, & Kitano, 2017 | Japan Sea Stickleback *Gasterosteus nipponicus* | default | 1280 | Assess mutational load |
| Yoshitomi et al., 2018 | Coxsackievirus (CV)‐A6 | not reported | 1 | Clinical symptoms |
| Zepeda Mendoza et al., 2018 | Vampire bat, *Desmodus rotundus* | default | At least 4 | Assess function of candidate variants for adaptation |
| Zhang, Zhou, Bawa, Suren, & Holliday, 2016 | Black cottonwood, *Populus trichocarpa* | not reported | approx. 20000 | Assess mutational load |
